# Estimation of radiation gonadal doses for the American–Ukrainian trio study of parental irradiation in Chornobyl cleanup workers and evacuees and germline mutations in their offspring

**DOI:** 10.1088/1361-6498/abf0f4

**Published:** 2021-11-01

**Authors:** Vadim Chumak, Elena Bakhanova, Victor Kryuchkov, Ivan Golovanov, Konstantin Chizhov, Dimitry Bazyka, Natalia Gudzenko, Natalia Trotsuk, Kiyohiko Mabuchi, Maureen Hatch, Elizabeth K Cahoon, Mark P Little, Tatiana Kukhta, Amy Berrington de Gonzalez, Stephen J Chanock, Vladimir Drozdovitch

**Affiliations:** 1 National Research Centre for Radiation Medicine, Kyiv, Ukraine; 2 Burnasyan Federal Medical and Biophysical Centre, Moscow, Russia; 3 Division of Cancer Epidemiology and Genetics, National Cancer Institute, NIH, DHHS, 9609 Medical Center Drive, Room 7E548 MSC 9778, Bethesda, MD 20892-9778, United States of America; 4 United Institute of Informatics Problems, Minsk, Belarus

**Keywords:** Chornobyl, cleanup worker, evacuee, radiation, dose, gonads

## Abstract

Radiation doses of parents exposed from the Chornobyl accident as cleanup workers or evacuees were estimated in the National Cancer Institute-National Research Center for Radiation Medicine trio (i.e. father, mother, offspring) study aimed at investigating the radiation effects on germline *de novo* mutations in children as well as other outcomes. Paternal (testes) and maternal (ovaries) gonadal doses were calculated along with associated uncertainty distributions for the following exposure pathways: (a) external irradiation during the cleanup mission, (b) external irradiation during residence in Pripyat, and (c) external irradiation and (d) ingestion of radiocesium isotopes, such as ^134^Cs and ^137^Cs, during residence in settlements other than Pripyat. Gonadal doses were reconstructed for 298 trios for the periods from the time of the accident on 26 April 1986 to two time points before the child’s date of birth (DOB): 51 (DOB-51) and 38 (DOB-38) weeks. The two doses, DOB-51 and DOB-38 were equal (within 1 mGy) in most instances, except for 35 fathers where the conception of the child occurred within 3 months of exposure or during exposure. The arithmetic mean of gonadal DOB-38 doses was 227 mGy (median: 11 mGy, range 0–4080 mGy) and 8.5 mGy (median: 1.0 mGy, range 0–550 mGy) for fathers and mothers, respectively. Gonadal doses varied considerably depending on the exposure pathway, the highest gonadal DOB-38 doses being received during the cleanup mission (mean doses of 376 and 34 mGy, median of 144 and 7.4 mGy for fathers and mothers, respectively), followed by exposure during residence in Pripyat (7.7 and 13 mGy for mean, 7.2 and 6.2 mGy for median doses) and during residence in other settlements (2.0 and 2.1 mGy for mean, 0.91 and 0.81 mGy for median doses). Monte Carlo simulations were used to estimate the parental gonadal doses and associated uncertainties. The geometric standard deviations (GSDs) in the individual parental stochastic doses due to external irradiation during the cleanup mission varied from 1.2 to 4.7 (mean of 1.8), while during residence in Pripyat they varied from 1.4 to 2.8 (mean of 1.8), while the mean GSD in doses received during residence in settlements other than Pripyat was 1.3 and 1.4 for external irradiation and ingestion of radiocesium isotopes, respectively.

## 1. Introduction

The U.S. National Cancer Institute (NCI) in collaboration with the National Research Center for Radiation Medicine (NRCRM) of the National Academy of Medical Sciences of Ukraine is conducting a study (NCI-NRCRM trio study) to investigate the transgenerational effects in the offspring of cleanup workers (liquidators) and evacuees (Bazyka *et al* 2020). Cleanup workers and evacuees are among those most exposed to external irradiation from the Chornobyl (Chernobyl) accident (UNSCEAR 2011), and, therefore, are supposedly the most appropriate subgroups of the population to be included in the study.

The NCI-NRCRM trio study sets a unique demand for assessing the gonadal doses of the parents of offspring accumulated during the preconception period. The unique features of this study included an independent estimation of doses to both parents (even presumably non-exposed), consideration of doses to the gonads (ovaries and testes) accumulated over a specific, individually defined period, and consideration of both external and internal components of Chornobyl-related exposure.

This paper describes the methodology developed and applied to evaluate individual doses and discusses the results of dose reconstruction within this unique trio study in Chornobyl cleanup workers and evacuees.

## 2. Materials and methods

### 2.1. Study population

A detailed description of the study design and methods can be found elsewhere (Bazyka *et al* 2020). In brief, the study included father–mother–offspring families (predominately trios). The parents (fathers and mothers) within the study were ascertained from the Clinical-Epidemiological Registry (CER) located at the NRCRM. Fathers and mothers represented five distinct exposure categories that were defined based on preliminary estimates of exposure available in the Chornobyl State Registry—‘State Registry of Ukraine’ (SRU) and the CER (Bazyka *et al* 2020):

- Category A: exposed father, unexposed mother. Exposed fathers typically were Chornobyl cleanup workers. Unexposed mothers typically resided outside the 30 km zone around Chornobyl Nuclear Power Plant (NPP);
- Category B: exposed mother, unexposed father. Exposed mothers typically were Chornobyl cleanup workers or residents of Pripyat town. Unexposed fathers typically resided outside the 30 km zone around Chornobyl NPP;
- Category C: both parents were exposed;
- Category D: both parents were unexposed; and
- Category E: father was a highly exposed first responder and/or an emergency cleanup worker, including those who developed acute radiation syndrome (ARS).

**Figure 1.**
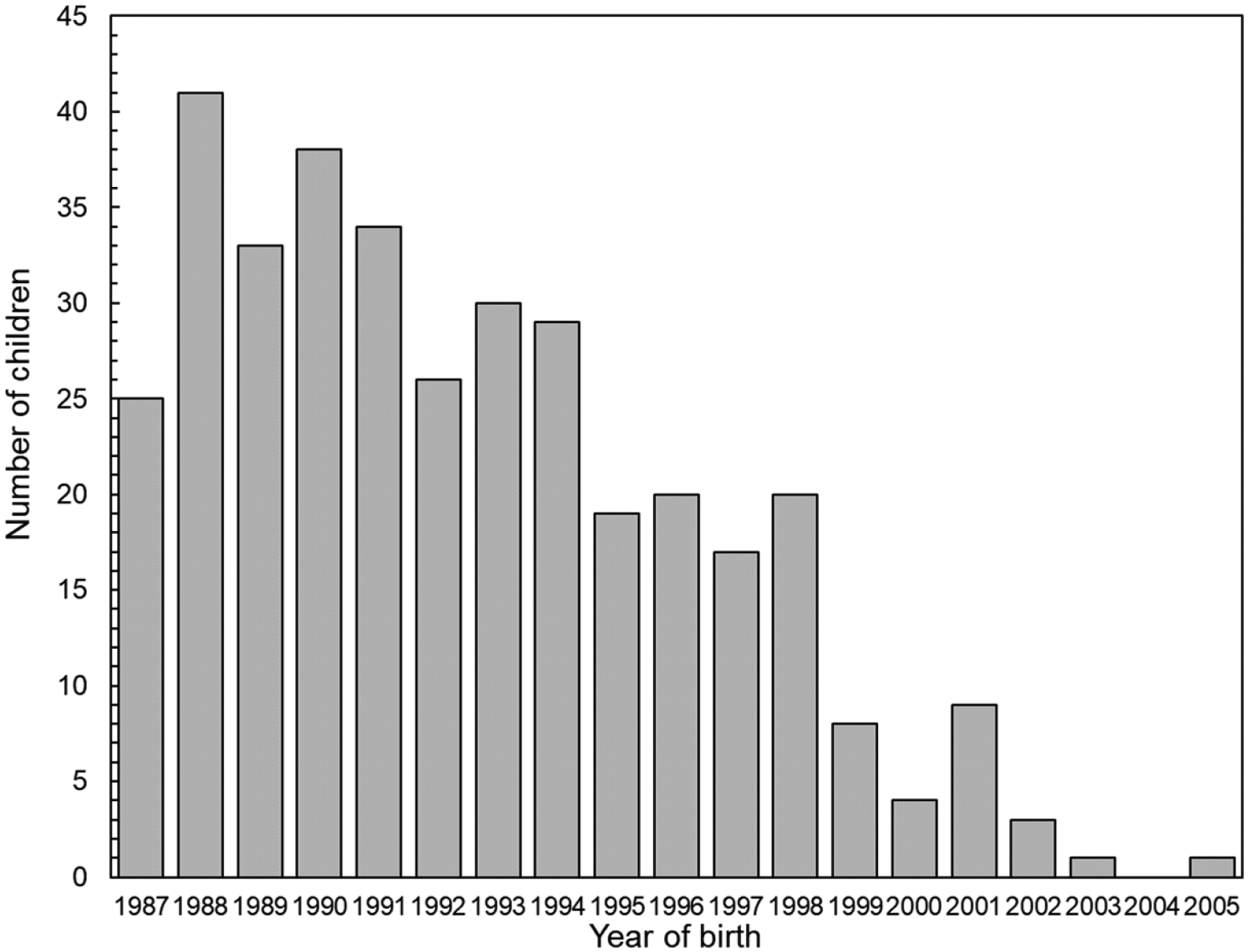
Distribution of children according to year of birth.

Only parents with offspring born 46 weeks or later after the Chornobyl accident were eligible for the current study. The number of offspring in a trio was not limited to one, so some ‘trios’ indeed included four or five members. Two hundred and ninety-eight complete trios were recruited, including 242 families with one child, 52 families with two children and four families with three children, for a total of 358 offspring, who were born between 14 March 1987 and 15 July 2005. Figure 1 shows the distribution of children according to year of birth. Almost 40% of them (137 out of 358) were born in 1990 or earlier when the main exposure occurred among Chornobyl cleanup workers and residents of contaminated territories.

It should be noted that 298 trios included in this study represent the entire planned study population that was recruited by February 2020. The papers, which were previously published for this study, included a subset of the full 298 trios: (a) 150 trios testing and describing the study methodology (Bazyka *et al* 2020) and (b) 105 trios analysing the genomic profile of transgenerational effects of ionizing radiation exposure achieved to date (Yeager *et al* 2021).

### 2.2. Personal interview

Both parents were interviewed in person by qualified interviewers between 26 October 2015 and 21 February 2020. To account for all potential components of radiation exposure from Chornobyl, the following three dosimetry questionnaires were administered to the parents (appendices A1–A3 (available online at stacks.iop.org/JRP/41/764/mmedia)):

- Mission as a Chornobyl cleanup worker. This questionnaire was designed to estimate the individual gonadal dose received during an individual’s work during the cleanup mission(s) and used to collect detailed information on (a) cleanup worker’s routes to and from his/her work location(s) at the Chornobyl site and in the 30 km zone, (b) cleanup activities he/she performed, including duration and applicable shielding from radiation and (c) locations of residence and resting quarters during the mission. This questionnaire was similar to the questionnaires administered to the subjects of the Ukrainian–American case-control studies of (1) leukaemia and related disorders (Chumak *et al* 2015) and (2) thyroid cancer among Chornobyl cleanup workers (Drozdovitch *et al* 2020). The questionnaire covers the entire period of participation in the Chornobyl cleanup activities between 26 April 1986 and 31 December 1990 (the technical date of the official completion of the Chornobyl cleanup).
- Residence in Pripyat. This questionnaire was used to collect information on (a) detailed hour-by-hour outdoor/indoor locations between the time of the accident and evacuation, (b) exact address in Pripyat and floor of the residence and (c) evacuation route within the 70 km zone. This questionnaire was a thoroughly revised version of the questionnaire used in the Ukrainian–American cohort study of thyroid cancer and other thyroid diseases (Likhtarov *et al* 2014). The questionnaire covers the time from the accident outbreak (1:23 AM on 26 April 1986) to the moment of eventual departure from the 70 km zone after evacuation.
- Residence at locations other than Pripyat. This questionnaire was specifically designed for this study to elicit information on (a) places and dates of residence(s) after the Chornobyl accident, (b) construction material of residential buildings in each place of residence and (c) consumption of locally produced foodstuff, namely milk, milk products, meat (pork, poultry), potatoes, root vegetables and mushrooms during residence at each location. The questionnaire covers the entire period from the time of the accident to the date of birth (DOB) of the youngest offspring included in the study, except for participation in the cleanup mission and residence in Pripyat.

The dosimetry interviewers were trained by cleanup workers and former staff members of the Chornobyl NPP, who are well-informed about the chronology of cleanup activities at the Chornobyl site and within the 30 km zone. These experienced interviewers previously conducted personal interviews with around 1600 individuals included in the Ukrainian–American case-control studies (Chumak *et al* 2015, Drozdovitch *et al* 2020). The senior interviewer, a former cleanup worker, provided coordination between the interviewers and ensured quality control of the completed study questionnaires.

### 2.3. Radiation dose reconstruction

All applicable exposure pathways were taken into consideration. Parents, who were cleanup workers, including those staff members of the Chornobyl NPP, were exposed predominantly to external radiation from radionuclide-contaminated buildings, indoor locations and ground surface. The inhalation of radionuclides during the ten day period of releases from the destroyed reactor was, according to data from UNSCEAR (2000), a minor pathway for gonadal exposure. Parents who resided in Pripyat town received doses due to external irradiation from airborne gamma-emitting radionuclides in radioactive clouds passing over the town and those deposited on the ground and buildings with no contribution of internal dose due to ingestion and very limited contribution due to inhalation. In turn, some parents, who were residents of the highly contaminated areas in northern Ukraine or southern Belarus, received gonadal doses at their places of residence from both external irradiation and internal irradiation due to consumption of locally produced food contaminated with ^134^Cs and ^137^Cs.

According to the study design, gonadal doses, to testes for fathers and ovaries for mothers, were reconstructed for the periods from 26 April 1986 (time of the accident) until two time points: 51 and 38 weeks before the child’s DOB, DOB-51 and DOB-38, respectively. The DOB-38 time point identified the period prior to conception of the offspring, while DOB-51 marked the point of 3 months preconception. The difference between the two dates was intended to develop a dose estimate for the 3 month period before conception, when there could be potential exposure to paternal spermatids, whereas the DOB-51 dose corresponds to a dose to spermatogonial stem cells. For maternal exposure, the only relevant time point was assumed to be DOB-38. Table 1 summarizes the characteristics of the components of gonadal dose that were calculated in the study.

The gonadal doses from external irradiation during the cleanup mission were reconstructed either until DOB-51 and DOB-38 or until the end of the parent’s cleanup activity or until 31 December 1990, which is the last date of cleanup activities according to Ukrainian law, whichever is earlier. If DOB-51 and DOB-38 time points were later than either the end of the parent’s cleanup activity or 31 December 1990, the parent’s gonadal doses due to cleanup activities, DOB-51 and DOB-38, were the same. The gonadal doses due to external irradiation related to residence in Pripyat were reconstructed until the moment of eventual departure from the 70 km zone in the course of evacuation between 27 April 1986, the date of evacuation of women and children, and the beginning of May 1986, when the Chornobyl NPP personnel were evacuated. The gonadal doses during residence in the settlements other than Pripyat were reconstructed until DOB-51 and DOB-38. Within the study cohort, the latest dose reconstruction date was 22 October 2004 for the youngest child in this study. The way individual gonadal doses due to different exposure pathways were estimated is described in the following section. The values of parameters of dose reconstruction models are given in section 2.4. ‘Assessment of uncertainties in gonadal dose estimates’.

#### 2.3.1. Gonadal doses due to external irradiation during cleanup mission.

A time-and-motion method named RADRUE was used previously to estimate external doses to cleanup workers (Kryuchkov *et al* 2009). This paper describes both the methodology and available input data and parameters of the model, which are used for the calculation of individual doses of cleanup workers. The RADRUE technique calculates dose due to external irradiation as a product of the air kerma rate, irradiation time and location factor (LF) that accounts for the shielding properties of buildings and the local environment. Gonadal absorbed dose during a cleanup mission was calculated as:

(1) $$D_{\text{ext}}^{\text{mission}}=C_{\text{g}}\cdot \sum_{i=1}^{N}\sum_{j=1}^{L_{i,j}}\text{AKR}\left(t_{i,j}\right)\cdot \Delta t_{i,j}\cdot {\text{LF}}_{j},$$

where $D_{\text{ext}}^{\text{mission}}$ is the absorbed gonadal dose due to external irradiation during the cleanup mission (mGy); *C*_g_ is the conversion coefficient from the air kerma rate to the absorbed dose rate in gonads. It was taken according to ICRP (1996) to be 0.710 for males and 0.586 for females for isotropic irradiation geometry for photon energy of 0.3 MeV (mGy h^−1^ per mGy h^−1^); *N* is the number of days of the cleanup mission; *L*_*i,j*_ is the number of cleanup activities at different locations *j* on day *i* considered in the calculation (usually unequal between different days of the cleanup mission). The considered activities cover time spent working, travelling, or resting during the cleanup mission; AKR (*t*_*i,j*_) is the air kerma rate at the location *j* of the cleanup mission on day *i* (mGy h^−1^); Δ*t*_*i,j*_ is the time interval of performing the complete cleanup activity at location *j* on day *i* (h); LF_*j*_ is the location factor at the place of cleanup activity *j* (unitless).

Detailed descriptions of the questionnaire data processing, data entry and validation that were applied in the RADRUE method can be found elsewhere (Kryuchkov *et al* 2009). It should be noted that the computer code originally developed to implement the RADRUE method has been modified for the purposes of the Ukrainian–American case-control study of thyroid cancer among Chornobyl cleanup workers, yielding a new computer code named Rockville (Drozdovitch *et al* 2020), which was also used in this study.

#### 2.3.2. Gonadal doses due to external irradiation related to residence in Pripyat.

Most of the residents of Pripyat were evacuated on the afternoon of 27 April 1986. By 30 April 1986, most of the Chornobyl NPP personnel, who still remained in Pripyat after evacuation of their families, were moved to the pioneer (youth) camp ‘Skazochny’ (Drozdovitch *et al* 2019). Ninety-three fathers and 88 mothers from the study resided in Pripyat at the time of the accident. A special methodology was developed and applied to assess the exposure of evacuees from Pripyat. The LF-values, i.e. ratio of the air kerma rate at a given exposure point to the air kerma rate at reference condition, i.e. undisturbed lawn far from any buildings, were calculated for each apartment in each building in Pripyat (indoor LF-values) as well as for locations outside buildings (outdoor LF-values). The LF-values for Pripyat were calculated with respect to exposure from radioactivity deposited on the ground surface and from the passing radioactive cloud.

The absorbed dose in the gonads due to external irradiation during residence in Pripyat was calculated using the RADRUE technique in the same way as for any other element of the cleanup mission:

(2) $$D_{\text{ext}}^{\text{Pripyat}}=C_{\text{g}}\cdot \sum_{i=1}^{N}\text{AKR}\left(t_{i}\right)\cdot \Delta t_{i}\cdot {\text{LF}}_{i},$$

where $D_{\text{ext}}^{\text{Pripyat}}$ is the absorbed gonadal dose due to external irradiation during residence in Pripyat (mGy); *N* is the number of different locations in Pripyat (apartment, store, outdoors, etc) where individuals spent time before the evacuation (unitless); AKR(*t*_*i*_) is the air kerma rate (mGy h^−1^) at location *i* in Pripyat at time *t*_*i*_; Δ*t*_*i*_ is the duration of staying in Pripyat at location *i* (h); LF_*i*_ is the location factor at location *i* (unitless).

The developed approach was implemented into the Rockville computer code for evacuees from Pripyat as a special module to calculate doses due to external irradiation. For the cleanup workers who resided in Pripyat, the dose received during residence was calculated together with the dose received during the cleanup mission. For operational reasons, it was inconvenient to separate these two components of exposure. In addition, there was no need for such separation as the total radiation dose from all components of exposure was required to be estimated.

#### 2.3.3. Gonadal doses during residence in the settlements other than Pripyat.

The majority of the parents resided in areas contaminated from Chornobyl fallout. Table 2 shows the distribution of the parent’s place of residence other than Pripyat between the time of the accident and DOB-38 and range of ^137^Cs deposition density in these locations. Among them, 140 parents resided in or visited highly contaminated settlement(s) with ^137^Cs deposition density of more than 555 kBq m^−2^ in northern Ukraine (134 persons) and in southern Belarus (six persons). One mother resided in the village of Kryuki in Gomel Oblast (Belarus), the most contaminated settlement after the Chornobyl accident with ^137^Cs deposition density of 18 530 kBq m^−2^.

External exposure of the population after the Chornobyl accident resulted from the ground deposits of a range of gamma-emitting radionuclides, including ^95^Zr, ^95^Nb, ^99^Mo, ^103^Ru, ^106^Ru, ^132^Te, ^131^I, ^132^I, ^133^I, ^134^Cs, ^136^Cs, ^137^Cs, ^140^Ba, ^140^La, ^141^Ce, ^144^Ce and ^239^Np (radionuclides are listed according to increasing mass number, not according to their amount, radiological significance or contribution to the dose). The approach for external dose calculation was based on the integration of the time-dependent dose rate in air per unit deposition of radionuclides, considering the shielding properties of the residential environment and individual behaviour of the person collected during the personal interview.

A detailed description of the applied model can be found elsewhere (Likhtariov *et al* 1996, Likhtarev *et al* 2002, Minenko *et al* 2006). In brief, the absorbed dose in gonads due to external irradiation during residence in the settlement other than Pripyat was calculated as:

(3) $$D_{\text{ext}}^{\text{resid}}={\text{BF}}_{m}\cdot \text{IV}\cdot \sum_{i}{\text{DC}}_{i}\cdot \int_{t_{1}}^{t_{2}}\sigma_{\text{Cs}137}\cdot R_{i/\text{Cs}137}\cdot {\text{e}}^{-\lambda_{i}^{r}\cdot t}\cdot p(t)\text{d}t,$$

where $D_{\text{ext}}^{\text{resid}}$ is the gonadal dose due to external irradiation during residence in the settlements other than Pripyat (mGy); ${\text{BF}}_{m}=\underset{n}{\sum }{\text{LF}}_{m,n}\cdot {\text{OF}}_{m,n}$ is the behaviour factor that takes into account the location factor at place *n* indoor or outdoor (LF_*m,n*_) and the typical fraction of time spent during the 24 h by a person at this place *n* (OF_*m,n*_) in a rural or urban *m*-th type of settlement (unitless); IV is a factor that accounts for individual variability in time spent outdoors (unitless); DC_*i*_ is the absorbed gonadal dose rate per unit activity of radionuclide *i* deposited on the ground surface (mGy d^−1^ per kBq m^−2^). Gender-specific DC_*i*_ values were taken from Jacob *et al* (1988); *σ*_Cs137_ is the ^137^Cs deposition density in the settlement of residence (kBq m^−2^); *R*_*i*/Cs137_ is the ratio of activity of radionuclide *i* in deposition to that of ^137^Cs (unitless). For radionuclides ^95^Nb, ^132^I and ^140^La that are decay products of ^95^Zr, ^132^Te and ^140^Ba the time-dependent deposition density was calculated taking into account the radioactive decay of both parental and daughter radionuclides; $\lambda_{i}^{r}$ is the radioactive decay rate of radionuclide *i* (d^−1^); *p*(*t*) is the attenuation function that reflects the decreasing dose rate due to radionuclide migration in the soil; it was applied for long-lived ^134^Cs and ^137^Cs only (unitless); *t*_1_, *t*_2_ are times corresponding to the beginning and end of staying in the settlement of residence (d).

The model to estimate doses due to the ingestion of radiocesium isotopes was based on the relationship between ^137^Cs deposition density and ^137^Cs soil-to-milk transfer and on the intake of radiocesium isotopes with foodstuffs. The intake derived from direct whole-body counting (WBC) measurements of radiocesium body burden was carried out in regions of Ukraine and Belarus with different contamination levels for populations of different ages. In brief, the absorbed dose in gonads due to the ingestion of radiocesium isotopes during residence in the settlement other than Pripyat was calculated as:

(4) $$D_{\text{ing}}^{\text{resid}}=\sigma_{\text{Cs}137}\cdot \sum_{k}{\text{DF}}_{k}\cdot \int_{t_{1}}^{t_{2}}I_{k}(t)\text{d}t,$$

where $D_{\text{ing}}^{\text{resid}}$ is the gonadal dose due to the ingestion of radiocesium isotopes during residence in the settlements other than Pripyat (mGy); *k* is the index to denote radiocesium isotopes, ^134^Cs and ^137^Cs; DF_*k*_ is the absorbed gonadal dose due to intake via ingestion of unit activity of radiocesium isotope *k* (mGy Bq^−1^) (ICRP 1993); *I*_*k*_ (*t*) is the variation with time of the intake function of radiocesium isotope *k* normalized to ^137^Cs deposition density in the settlement of residence (Bq d^−1^ per kBq m^−2^).

With respect to the specifics of foodstuff contamination, two time periods were considered. The first period began at the time of the accident and lasted until 31 July 1986; during this period the contamination of vegetation and, consequently, of cow’s milk, milk products and meat (pork, poultry) was caused mainly by radionuclides deposited on the grassland surface. From 1 August 1986 onwards, the root uptake of radionuclides by vegetation became more important. In addition to the foodstuffs indicated above, the consumption of potatoes, root vegetables and mushrooms was considered. WBC measurements were used for residents of rural settlements to validate their intake function of radiocesium isotopes with locally produced foodstuffs, *I*_*k*_ (*t*). For residents of mixed rural–urban and urban types of settlements, doses due to the ingestion of radiocesium isotopes were directly derived from WBC measurements. A detailed description of the model and estimates of intake function can be found elsewhere (Likhtarev *et al* 1996, 2000, Minenko *et al* 2006).

The dose received during the residence in contaminated areas was calculated as the sum of doses due to external irradiation (equation (3)) and ingestion of radiocesium isotopes (equation (4)) received in all locations of residence in the period between the time of the accident and DOB-51/DOB-38.

### 2.4. Assessment of uncertainties in gonadal dose estimates

Individual stochastic parental doses were calculated for each child: 10 000 doses due to external irradiation during the cleanup mission and residence in Pripyat, and 1000 doses due to external irradiation and ingestion of radiocesium isotopes during the residence in settlements other than Pripyat. The distribution of individual stochastic doses was found to be typically log-normal and the geometric standard deviation (GSD) was used to characterize the uncertainty of the doses (e.g. Kryuchkov *et al* 2009, Drozdovitch *et al* 2020). The Monte Carlo simulation technique was used to estimate the uncertainties in each of the parental dose components, which are described in sections 2.3.1–2.3.3 above.

The RADRUE method and the Rockville computer code facilitate the estimation of dose and its uncertainties associated with uncertainties of the input parameters. The parameters of the RADRUE model and their distribution used to calculate 10 000 individual stochastic gonadal doses are given in table 3. All parameters were associated with unshared errors as the ‘shared’ dose caused by the situation when cleanup workers were sharing the same location at the same time represents less than 1% of the total dose (Kryuchkov *et al* 2009).

Detailed information on hour-by-hour occupancy in outdoor/indoor locations between the time of the accident and evacuation was collected by means of a personal interview and the LF-value during different time intervals and points of exposure of the person, were used in the Rockville computer code to calculate 10 000 individual stochastic doses for the residents of Pripyat.

For exposure during residence in settlements other than Pripyat, the 2D Monte Carlo method, which accounts for shared and unshared errors (UNSCEAR 2014, Simon *et al* 2015, NCRP 2018), was used in this study to estimate uncertainties associated with the gonadal doses. According to this approach, 1000 sets of the study population gonadal doses were calculated for each exposure pathway: external irradiation and ingestion of radiocesium isotopes. For a specific dose realization, some of the model parameter values were common among members of subgroups, i.e. shared among persons of those groups, implying that any error made on this parameter was shared by all individuals to whom it was applied. Subject-independent or shared parameters of the model are given in table 4. Other uncertainties were subject dependent or unshared including errors related to the individual foodstuff consumption reported during the personal interview (table 5). At the beginning of the calculation of each dose set for the entire study population, values for all shared parameters were assigned. The same value for each shared parameter was used to calculate one dose set for all individuals for whom this parameter was shared. In the process of stochastic dose simulation, the values of unshared parameters for each person were sampled from their distributions and one dose realization was calculated for the entire study population.

The 10 000 (for doses during the cleanup mission and residence in Pripyat) or 1000 (for doses received during residence in other settlements) realizations of dose for an individual represent *the individual stochastic gonadal doses* assessed for this person. Each stochastic estimate may be assumed to be sampled from the true dose distribution. An arithmetic mean of 10 000 or 1000 individual stochastic dose values was used as the central estimate of the individual gonadal dose due to a specific exposure pathway. The individual paternal or maternal gonadal dose was calculated by summing the arithmetic mean doses for all four exposure pathways.

## 3. Results

### 3.1. Gonadal dose estimates

Table 6 provides parental gonadal DOB-38 doses per each child from all exposure pathways by exposure category used to ascertain the trios. For most of the subjects the two doses concerned, DOB-51 and DOB-38, differed by less than 1 mGy, except for 35 fathers with a difference between the two doses of more than 1 mGy (maximal difference = 260 mGy) because the conception of the child occurred within the 3 month period after exposure or during the exposure period. A cross-classification of parental DOB-51 and DOB-38 doses (table 7) shows that for only six children, the paternal gonadal dose moved to a higher dose category due to the radiation exposure occurring between the dates of DOB-51 and DOB-38. There were no changes for the maternal DOB-51 and DOB-38 gonadal dose between dose categories as the difference between these two doses was less than 1 mGy (maximal difference = 0.55 mGy). One of the reasons for this miniscule discrepancy is fluctuation in the stochastic simulation leading to slight modification of the arithmetic mean of individual stochastic doses.

The arithmetic mean of gonadal DOB-38 doses across the study (358 children) was 227 mGy (median of 11 mGy, range 0–4080 mGy) and 8.5 mGy (median of 1.0 mGy, range 0–550 mGy) for fathers and mothers, respectively. The means and ranges calculated separately for children falling into the particular category (table 6) varied considerably by exposure category, with the highest doses observed in category E (high-dose emergency workers) having a mean dose of 596 mGy (median of 254 mGy) and 17 mGy (median of 0.72 mGy) for fathers and mothers, respectively, followed by category C (both parents exposed), having a mean dose of 322 mGy (median of 63 mGy) and 16 mGy (median of 7.1 mGy) for fathers and mothers, respectively.

Gonadal doses varied considerably by exposure pathway (tables 8 and 9). For external irradiation during the cleanup mission, the average parental gonadal DOB-38 doses for fathers and mothers who were involved in cleanup activities were estimated to be 376 mGy (median of 144 mGy, range 0.063–4080 mGy) and 34 mGy (median of 7.4 mGy, range 0.013–550 mGy), respectively. The average gonadal DOB-38 doses from external irradiation during residence at Pripyat were estimated to be 7.7 mGy (median of 7.2 mGy, range 3.1–17 mGy) for fathers and 13 mGy (median of 6.2 mGy, range 0.21–260 mGy) for mothers. The average gonadal DOB-38 doses for fathers and mothers during residence in contaminated areas other than Pripyat were 2.0 mGy (median of 0.91 mGy, range 0–48 mGy) and 2.1 mGy (median of 0.81 mGy, range 0–51 mGy), respectively. For 242 children (67.6% of the total) the paternal gonadal DOB-38 doses and for 355 children (99.2%) the maternal gonadal DOB-38 doses from all exposure pathways combined were less than 100 mGy.

**Figure 2.**
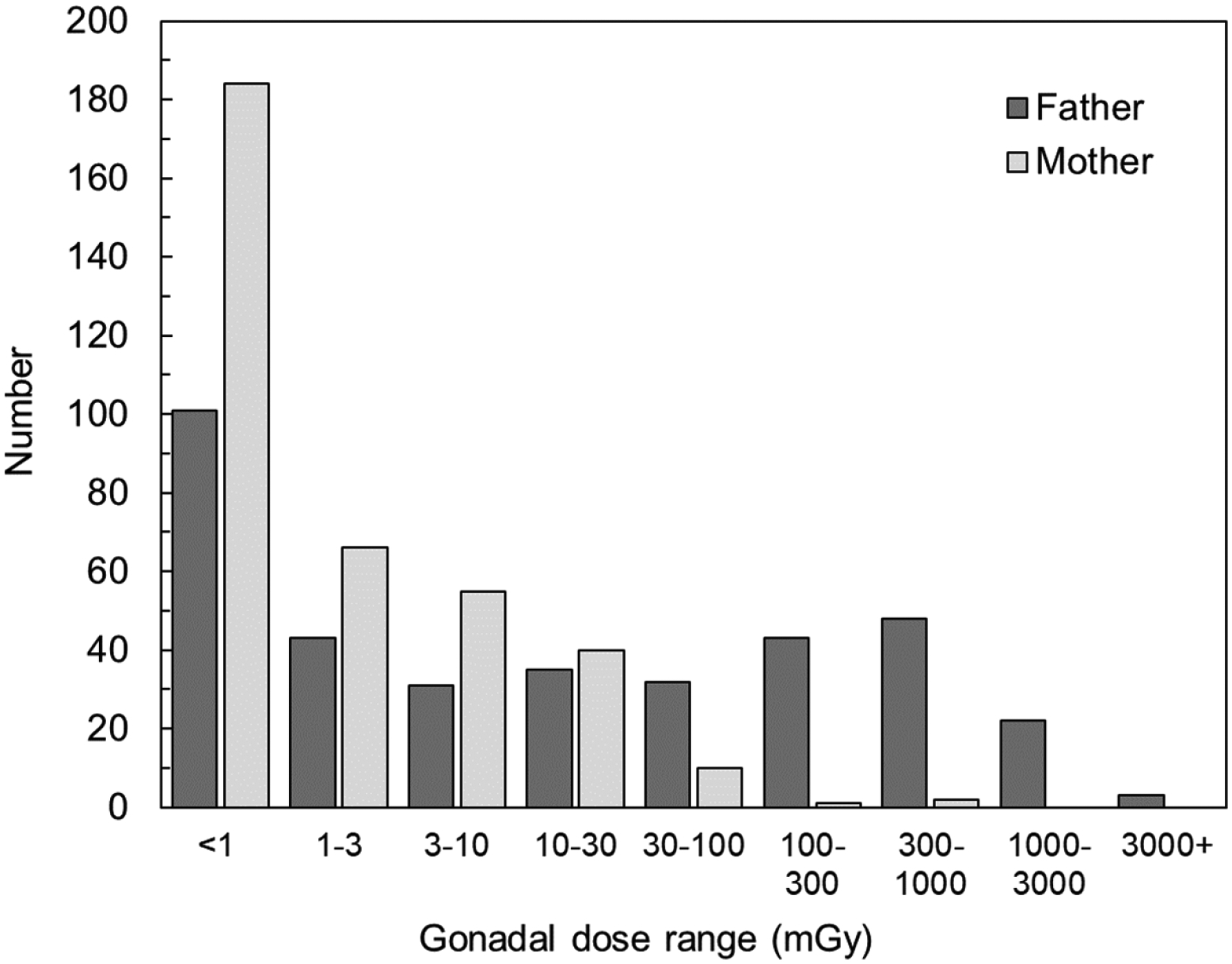
Distribution of children according to paternal and maternal gonadal DOB-38 doses from all exposure pathways.

Figure 2 shows the overall distribution of children according to the father’s and mother’s gonadal DOB-38 doses from all exposure pathways. For 175 children (48.9% of the total) the father’s gonadal dose and for 305 children (85.2%) the mother’s gonadal doses were estimated to be less than 10 mGy. For 25 study children (7.0%) the father’s gonadal dose was 1000 mGy or more.

There is a negative correlation between the paternal gonadal doses and the year of birth of the child with Pearson correlation coefficient, *r*_*p*_ = −0.23 (*p* < 0.001) for the logarithm of the dose values and *r*_*p*_ = −0.13 (*p* = 0.0156) for the linear dose values. There is a weak trend between the maternal gonadal doses and the year of birth of the child with Pearson correlation coefficient, *r*_*p*_ = 0.049 (*p* = 0.358) for the logarithm of the dose values and *r*_*p*_ = −0.029 (*p* = 0.592) for the linear dose values (not shown).

Table 10 shows the distribution of the father’s and mother’s gonadal DOB-38 doses due to external irradiation during the cleanup mission by category of cleanup workers. It should be noted that (a) the doses are given for fathers and mothers, not for children; and (b) the doses were calculated only until 38 weeks before the child’s DOB and, therefore, do not represent the entire history of radiation exposure during the cleanup mission for some parents. If a father or mother has two or three children, the highest paternal dose, which reflects longer exposure history, was included in the table. For one father, who was at the Chernobyl NPP at the time of the accident and who was later diagnosed with ARS (falls into the category ‘victim of the accident’), the gonadal dose was 4080 mGy. The highest exposure categories of male cleanup workers included individuals who belonged to the following categories: AC-605 staff, i.e. persons from the specialized organization, named Administration of Construction No. 605, who were involved in the construction of the shelter covering the damaged reactor (mean dose = 746 mGy), sent to assist the Chornobyl NPP staff (543 mGy) and those who worked at the Chornobyl site several times as members of different categories, the ‘mixed’ group in table 10 (538 mGy). The highest mothers’ gonadal doses were estimated for the ‘mixed’ category with mean dose of 39 mGy and median of 9.3 mGy.

### 3.2. Uncertainties in gonadal doses

Table 11 shows the distributions of the GSDs attached to the individual stochastic gonadal doses DOB-38 from different exposure pathways for both parents combined. The GSDs in the parental individual stochastic doses due to external irradiation during the cleanup mission varied from 1.2–4.7 with a mean equal to 1.8. For more than half of the children (139 out of 260) the GSDs of the parental individual stochastic doses during the cleanup mission varied between 1.5–2.0. The largest GSDs (>2.5) were linked with the highest doses and were associated with uncertainties in the estimates of exposure rate grids at the Chornobyl site and with information on the exact duration and location of the cleanup mission tasks that were reported by a father or mother during the personal interview. The GSDs in the individual stochastic doses received during residence in Pripyat varied from 1.4–2.8 with a mean of 1.8. The mean GSD in the individual stochastic doses received during residence in settlements other than Pripyat was 1.3 and 1.4 for external irradiation and ingestion of radiocesium isotopes, respectively. Uncertainties in the air kerma rate values, which were considered here, are related to interpolated values and take into account not only measurement errors, but the uncertainty in the interpolation of measured air kerma rate values in time and space. As such, these errors are a mixture of Berksonian and classical form and (as we discuss below) are largely unshared.

## 4. Discussion

This paper describes a comprehensive dose reconstruction done for the American–Ukrainian trio study of parental irradiation in Chornobyl cleanup workers and evacuees and germline mutations in their offspring. Overall, individual gonadal doses due to the main pathways of exposure were estimated for 298 trios for the periods from 26 April 1986 (time of the accident) to two time points: 51 and 38 weeks before the child’s DOB. As expected, the highest doses were received during the cleanup mission, which confirms our earlier findings on the predominant role of external irradiation in the exposure to the Ukrainian Chornobyl cleanup workers (Drozdovitch *et al* 2020).

**Figure 3.**
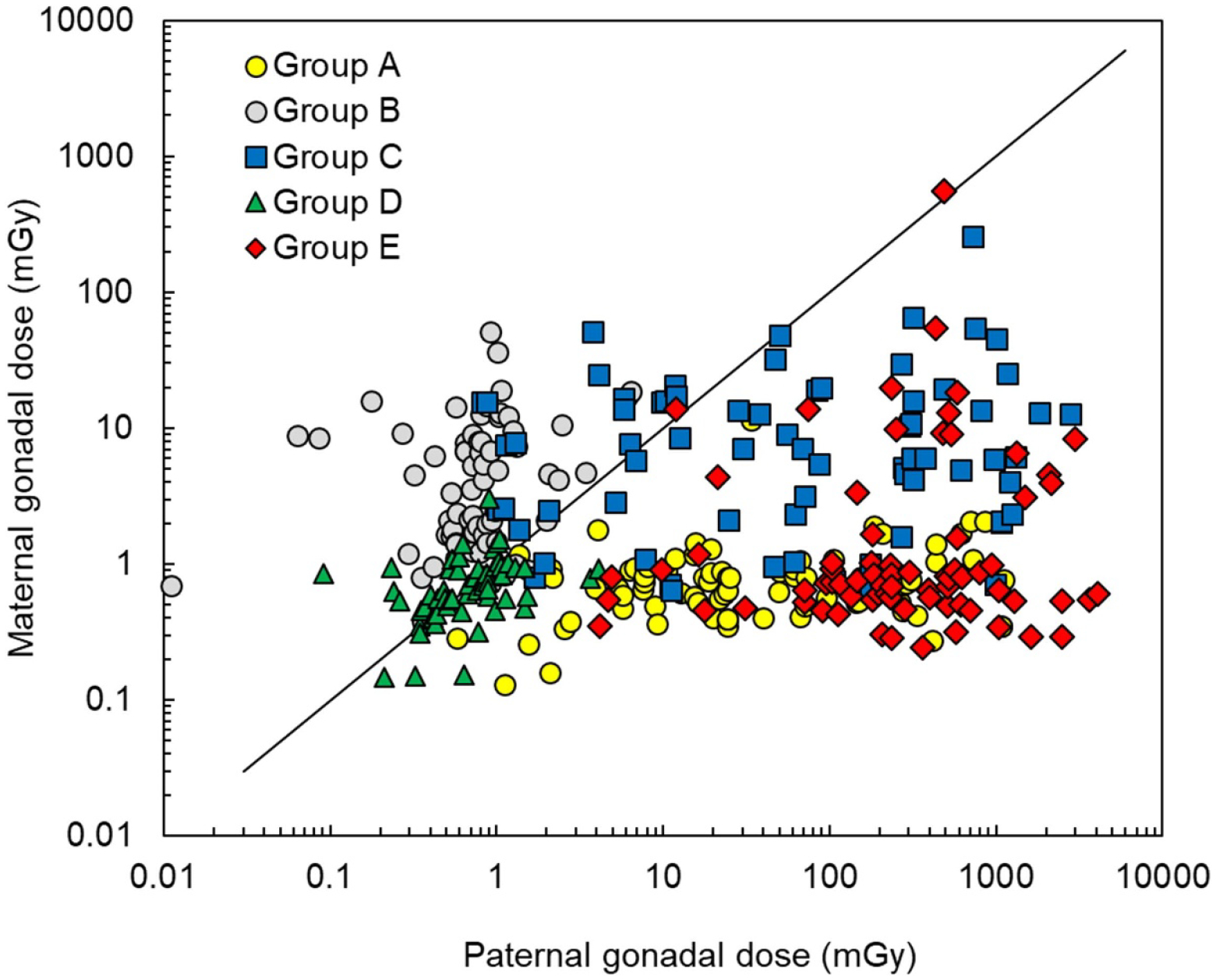
Correlation of paternal and maternal gonadal DOB-38 doses estimated in this study in relation to the initial exposure categories used to ascertain the trios.

The mean and range of dose estimates also varied by exposure category used to ascertain the trios, with the highest doses observed in category E ‘High-dose emergency workers’. However, for some trios, the exposure category, which was initially assigned based on official doses from the SRU or on doses available at the CER turned out to be incorrect and, after detailed dose reconstruction done in this study, around 25% of the trios moved from one exposure category to another. Figure 3 shows the correlation between paternal and maternal gonadal doses estimated in this study in relation to the initial exposure categories used to ascertain the trios. As can be seen from the figure, trios with maternal gonadal dose of a few mGy cannot be included in category C ‘Both parents were exposed’, as well as trios with paternal gonadal dose of a few mGy that cannot belong to category A ‘Exposed father, unexposed mother’. However, changes in the exposure categories would not compromise the study in any way as the epidemiological analysis uses continuous gonadal dose regardless of initial categorization (Yeager *et al* 2021). It is important to note that these discrepancies between official doses and doses estimated in this study using advanced detailed dosimetric techniques highlight the widely recognized problems with direct use of the official doses for epidemiological studies, although they can be useful in the planning stage of selecting study subjects.

Paternal and maternal mean gonadal doses were similar for exposure during residence in Pripyat, 7.7 and 13 mGy, respectively, and during residence in other settlements, 2.0 and 2.1 mGy, respectively. However, the mean doses due to external irradiation during the cleanup mission differ significantly between fathers and mothers, 376 versus 34 mGy, respectively (tables 8 and 9). The reason for much lower doses for female cleanup workers was explained by Chumak *et al* (2018) as being due to the difference in locations and respective types of work between male and female cleanup workers as the proportion of male cleanup workers who worked at the Chornobyl NPP site and within the 4 km zone was much higher than female cleanup workers.

Dose outliers were carefully considered for accuracy. Special attention was paid to a mother with an unusually high gonadal dose of 260 mGy from external irradiation during residence at Pripyat. This dose is 20 times higher than the mean dose in the group of female residents of Pripyat (13 mGy). During a personal interview, this woman reported that on 26 April 1986 she left Pripyat to visit parents in the town of Chornobyl. Since the bus service between towns was disrupted due to the accident, she walked and waited for a passing car for around 40 min in the immediate vicinity of the Chornobyl NPP. Therefore, she received a dose due to external irradiation comparable to the mean dose in the group of male cleanup workers (376 mGy).

The RADRUE method, which was used in this study to calculate gonadal doses due to external irradiation during the cleanup mission, i.e. the major contributor to parental doses, was also used in other epidemiological studies among Chornobyl cleanup workers to provide reliable individual dose estimates (e.g. Kesminiene *et al* 2008, 2012, Romanenko *et al* 2008, Chumak *et al* 2015, Cahoon *et al* 2019, Drozdovitch *et al* 2020). The strengths of this method are (a) the extensive validation of RADRUE-estimated doses using dose estimates based on thermoluminescence dosimeter and electron paramagnetic resonance in tooth enamel measurements and on quantitative analysis of unstable chromosome aberrations (dicentrics), (b) the availability of detailed data on cleanup workers’ activities collected by trained interviewers during a personal interview and (c) the involvement of dosimetry experts to interpret the data and provide additional information based upon their unique Chornobyl experience (Kryuchkov *et al* 2009).

However, the RADRUE method has some limitations. First, the resulting dose estimates may be distorted by ‘human factor’ uncertainties, that is to say, errors due to failure to adequately recall and answer questions about events that occurred in the distant past. The second limitation is related to the ‘intrinsic’ uncertainty in the estimates of air kerma rates at the sites of cleanup workers’ activities and in Pripyat, in part reflecting problems in measurement at particular locations at the time. Another possible limitation is related to the approach in assigning the parameters to be characterized by either shared or unshared errors. Although there is a shared error in the interpolation of measured air kerma rate values in time and space, we considered the air kerma rate uncertainty to be an unshared error. According to Kryuchkov *et al* (2009), less than 1% of the doses received during an entire cleanup mission was defined by the air kerma rate shared at the location where two or more cleanup workers were at the same time. Therefore, it was not deemed necessary to consider the shared component of uncertainty in the air kerma rate.

Nevertheless, in the absence of individual-based radiation doses for cleanup workers obtained using physical or biological dosimetry techniques, RADRUE appears to be the most appropriate method due to its simple applicability and conceptually clear analysis of the results.

## 5. Summary and conclusion

Individual gonadal doses to 298 fathers and 298 mothers of 358 children were estimated in the study of transgenerational effects in offspring of Chornobyl cleanup workers and evacuees from Pripyat. Parental gonadal doses were calculated for various time intervals before conception and for different exposure pathways, including external irradiation during the cleanup mission and residence in Pripyat town as well as external irradiation and the ingestion of radiocesium isotopes during residence in settlements other than Pripyat town. Although gonadal doses were reconstructed for the periods from the time of the accident on 26 April 1986, up to two time points: 51 and 38 weeks before the child’s birth, these two doses, DOB-51 and DOB-38, were on most occasions virtually equal (within 1 mGy) because conception of only 35 children occurred within the 3 month period after exposure or during exposure. The arithmetic mean of the gonadal DOB-38 doses was 227 and 8.5 mGy and the median was 11 and 1.0 mGy for fathers and mothers, respectively. Gonadal doses varied considerably by exposure pathway, with the highest doses being received during the cleanup mission, with mean DOB-38 doses of 376 mGy (median of 144 mGy) and 34 mGy (median of 7.4 mGy) for fathers and mothers, respectively, followed by exposure during residence in Pripyat, with mean DOB-38 doses of 7.7 mGy (median of 7.2 mGy) and 13 mGy (median of 6.2 mGy), respectively, and during residence in other settlements, with mean DOB-38 doses of 2.0 mGy (median of 0.91 mGy) and 2.1 mGy (median of 0.81 mGy) for fathers and mothers, respectively. The means and ranges of dose estimates also varied by exposure category, with the highest doses observed in category E ‘High dose emergency workers’, with mean paternal and maternal doses of 596 mGy (median of 254 mGy) and 17 mGy (median of 0.72 mGy), respectively, followed by category C ‘Both parents exposed’, with mean doses of 322 mGy (median of 63 mGy) and 16 mGy (median of 7.1 mGy) for fathers and mothers, respectively. Gonadal doses presented in this paper are being used to evaluate transgenerational effects of ionizing radiation exposure in cleanup workers and evacuees of the Chornobyl accident within the NCI-NRCRM trio study.

## Supplementary Material

Appendix 3

Appendix 1

Appendix 2

## Figures and Tables

**Table 1.** Characteristics of components of gonadal doses calculated for the parents.

Component	Pathway of exposure	Time frame of exposure	Exposure occurred at
External	Gamma-emitting radionuclides	Cleanup mission between 26 April 1986 and DOB-51/DOB-38 or 31 December 1990, if child was born after that date	Chornobyl NPP and the 30 km zone
External	Gamma-emitting radionuclides	Residence in Pripyat from 26 April 1986 until evacuation (27 April–early May 1986)	Pripyat
External	Gamma-emitting radionuclides	Residence between 26 April 1986 and DOB-51/DOB-38 (22 October 2004 for the youngest child included in the study)	Settlement of residence other than Pripyat
Internal	Intake of ^134^Cs and ^137^Cs with locally produced foodstuffs	Residence between 26 April 1986 and DOB-51/DOB-38 (22 October 2004 for the youngest child included in the study)	Settlement of residence other than Pripyat

**Table 2.** Distribution of the parents according to the places of residence other than Pripyat between the time of the accident and DOB-38 and the range of ^137^Cs deposition densities in these locations.

Country	Oblast/city	*N* ^a^	Deposition density of ^137^Cs (kBq m^−2^)
Ukraine	Kyiv-city	373	26
	Kyiv Oblast	299	3.0–6040
	Chernihiv Oblast	35	1.7–155
	Zhytomir Oblast	34	3.0–380
	Other oblasts	225	1.4–120
Belarus	Gomel Oblast	18	12–18 530
	Other oblasts	8	1.7–95
Russia		82	0–62
Other countries		41	—

a Total number of the parents does not equal to 596 as some of them resided in more than one location during the post-accident period.

**Table 3.** Parameters of the RADRUE model and their distributions used to calculate individual stochastic gonadal doses due to external irradiation considered to be unshared (subject dependent).

Parameter					
Description	Symbol (equation (1))	Unit	Central value (arithmetic mean (AM))	Distribution^c^	Reference
Duration of cleanup mission task	Δ*t*_*i,j*_	d	AM and CV^a^ from DB^b^	U((l − CV) × AM, (1 + CV) × AM) or U(0.9 × AM, 1.1 × AM)	Kryuchkov *et al* (2009)
Air kerma rate	AKR (*t*_*i,j*_)	mGyh^−1^	AM and GSD from DB	TLN(GM^d^, GSD, GSD^−2^ × GM, GSD^2^ × GM)	Kryuchkov *et al* (2009)
Conversion coefficient from air kerma rate to the gonadal absorbed dose rate	*C*_*g*_	mGy h^−1^ per mGyh^−1^	0.710 (male) 0.586 (female)	TN(AM, 0.1 × AM, AM − 2 × SD, AM + 2 × SD)	ICRP (1996)
Location factor for the Chornobyl site during the cleanup mission (equation (1))	*LF*_*j*_	Unitless	Table 2 from Kryuchkov *et al* (2009)	TN(AM, 0.25 × AM, AM − 2 × SD, AM + 2 × SD)	Kryuchkov *et al* (2009)
Location factor for residence in Pripyat (equation (2))	*LF*_*j*_	Unitless	0.072/0.0018/0.049^e,f^ 0.047/0.0012/0.0322^g^ 0.038/0.0001/0.025^h^	TLN(GM^d^, 1.3, 0.6 × GM, 1.7 × GM)	—^i^

a Coefficient of variation, CV = AM/SD.

b DB = database.

c U(min, max): uniform distribution with the following parameters: minimal value (min), maximal value (max). TLN(GM, GSD, min, max): truncated lognormal distribution with the following parameters: geometric mean (GM), geometric standard deviation (GSD), minimal value (min), maximal value (max). TN(AM, SD, min, max): truncated normal distribution with the following parameters: arithmetic mean (AM), standard deviation (SD), minimal value (min), maximal value (max). TR(min, mode, max): triangular distribution with the following parameters: minimal value (min), mode of distribution (mode), maximal value (max).

d $\text{GM}=\text{AM}\cdot {\left[\sqrt{\text{exp}\left({\left(\text{lnGSD}\right)}^{2}\right)}\right]}^{-1}$, derived from Carroll *et al* (2006); GM: geometric mean; AM: arithmetic mean; GSD: geometric standard deviation.

e LF-values for a five-storey building surrounded by several neighbouring buildings located at a distance of 75 m (a median of distance between buildings in the 1^st^ microdistrict in Pripyat). Values are given for the daytime/sleeping in the apartment during the night/indoor daily averaged, assuming 8 h sleeping.

f 1st floor of the building.

g 3rd floor of the building.

h 5th floor of the building.

i Chizhov, personal communication.

**Table 4.** Parameters of dosimetry model for residential exposure in settlements other than Pripyat considered to be shared (subject independent).

Parameter		Cental value (AM)^a^	Distribution^b^	Shared among subjects	Reference
Description	Unit				
^137^Cs ground deposition density in the settlement	kBq m^−2^	DB		The same settlement	
—Ukraine		—	TLN(0.96 × AM, 1.4, 0.5 × GM, 2.0 × GM)		Talerko (2005)
—Belarus		<185	TLN(0.96 × AM, 1.4, 0.5 × GM, 2.0 × GM)		Drozdovitch *et al* (2002)
—Belarus		⩾185	TLN(0.9 × AM, 1.6, 0.4 × GM, 2.6 × GM)		Drozdovitch *et al* (2002)
—Russia		—	TLN(0.9 × AM, 1.6, 0.4 × GM, 2.6 × GM)		Stepanenko *et al* (2004)
*External exposure during residence*					
Ratio of activity of radionuclide in deposition to that of ^137^Cs on 26 April 1986	Unitless	DB		The same area	Savkin *et al* (1996), Likhtarev *et al* (2002), Minenko *et al* (2006), Khrushchinskii *et al* (2014)
—^95^Zr, ^95^Nb^c^, ^141^Ce^d^, ^144^Ce^e^		—	TR (0.8 × AM, AM, 1.2 × AM)		
—^99^Mo, ^103^Ru, ^106^Ru^f^, ^140^Ba^g^, ^140^La, ^239^Np		—	TR(0.75 × AM, AM, 1.25 × AM)		
—^131m^Te, ^132^Te, ^131^I, ^132^I^h^, ^133^I^i^, ^135^I^i^		—	TR(0.85 × AM, AM, 1.15 × AM)		
—^134^Cs^j^, ^136^Cs^j^		—	—		
Behaviour factor for residence in	Unitless	DB		All	Meckbach and Jacob (1988), Golikov *et al* (2002)
—wooden house		0.34, 0.28^k^	TR(0.8 × AM, AM, 1.2 × AM)		
—brick or block single-floor house		0.24, 0.21	TR(0.8 × AM, AM, 1.2 × AM)		
—brick or block many-floor building		0.15, 0.13	TR(0.7 × AM, AM, 1.3 × AM)		
Attenuation function	Unitless	0.4	U(0.35,0.45)	All	Likhtarev *et al* (2002)
	Unitless	0.6	*p*_2_ = l – *p*_1_		
	d^−1^	1.27 × 10^−3^	TR(1.17 × 10^−3^, 1.39 × 10^−3^)		
	d^−1^	3.8 × 10^−5^	TR(3.2 × 10^−5^, 4.7 × 10^−5^)		
*Internal exposure during residence*					
Conversion factor of ^137^Cs activity for foodstuff in relation to ^137^Cs activity in private cow’s milk	Unitless	DB		All	Minenko *et al* (1996), Likhtarev *et al* (2000)
—Milk from shop		—	U(0.9, 1)		
—Milk products		—	U(0.5, 0.9)		
—Meat		—	U(0.8, 1)		
—Potato, root vegetables		—	U(0.12, 0.24)		
—Mushrooms		—	U(5, 15)		
*Dose in 1986*					
Concentration of ^137^Cs in private cow’s milk normalised to the ^137^Cs deposition density	Bq L^−1^ per kBq m^−2^	DB	TR(0.7 × AM, AM, 1.3 × AM)	All	Expert judgment
*Dose in 1987*+					
Relative concentration of ^137^Cs normalised to the ^137^Cs deposition density per unit of soil-to-milk ^137^Cs transfer	Unitless	DB	TR(0.7 × AM, AM, 1.3 × AM)	All	Expert judgment
Soil-to-milk transfer coefficient in the settlement	Bq L^−1^ per kBq m^−2^	DB	TR(0.6 × AM, AM, 1.4 × AM)	The same settlement	Minenko *et al* (1996)

a AM = arithmetic mean.

b TLN(GM, GSD, min, max): truncated lognormal distribution with the following parameters: geometric mean (GM), geometric standard deviation (GSD), minimal value (min), maximal value (max). TR(min, mode, max): triangular distribution with the following parameters: minimal value (min), mode of distribution (mode), maximal value (max). U(min, max): uniform distribution with the following parameters: minimal value (min), maximal value (max).

c Correlation (*r* = 0.95) with ratio for ^95^Zr.

d Correlation (*r* = 0.9) with ratio for ^144^Ce.

e Correlation (*r* = 0.95) with ratio for ^95^Zr.

f Correlation (*r* = 0.9) with ratio for ^103^Ru.

g Correlation (*r* = 0.95) with ratio for ^140^La.

h Correlation (*r* = 0.95) with ratio for ^132^Te.

i Correlation (*r* = 0.9) with ratio for ^131^I.

j Correlation (*r* = 1.0) with ^137^Cs deposition density.

k 30 km zone, other areas.

**Table 5.** Questionnaire data and parameters of dosimetry model for residential exposure considered to be unshared (subject dependent).

Parameter	Unit	Central value (arithmetic mean (AM))	Distribution^a^	Reference
*Consumption rate during residence*				
Consumption rate of foodstuff reported during the personal interview	kg d^−1^	Questionnaire DB	TR(0.75 × AM, AM, 1.25 × AM)	Drozdovitch *et al* (2015)
*External exposure during residence*				
Factor that accounts for individual variability in time spent outdoors	Unitless	1.0	U(0.7, 1.3)	Expert judgment
*Internal exposure during residence*				
Dose factors for gonads for ^134, 137^Cs ingestion	mGy Bq^−1^	DB	TLN(0.4 × AM, AM, 1.7 × AM)	ICRP (1993)
Consumption rate of mushrooms	kg d^−1^	If answer ‘yes’	U(0.010, 0.030)	Minenko *et al* (1996)

a TR(min, mode, max): triangular distribution with the following parameters: minimal value (min), mode of distribution (mode), maximal value (max). TLN(GM, GSD, min, max): truncated lognormal distribution with the following parameters: geometric mean (GM), geometric standard deviation (GSD), minimal value (min), maximal value (max). U(min, max): uniform distribution with the following parameters: minimal value (min), maximal value (max).

**Table 6.** Parental gonadal DOB-38 doses (mGy) from all exposure pathways by exposure category averaged by the number of children in the category.

			Gonadal DOB-38 dose^a^ (mGy) from all exposure pathways^b^ for					
			Paternal			Maternal		
Exposure category	Number of trios	Number of children	Arithmetic mean	Median	Range	Arithmetic mean	Median	Range
A (father exposed)	71	82	151	24	0.37–1120	0.89	0.73	0–12
B (mother exposed)	51	70	1.0	0.79	0–6.4	6.9	4.4	0.38–51
C (both exposed)	59	71	322	63	0.81–2790	16	7.1	0.64–260
D (both unexposed)	51	58	0.80	0.66	0–4.1	0.74	0.64	0.15–3.0
E (high-dose workers)	66	77	596	254	4.2–4080	17	0.72	0–550
Entire study	298	358	227	11	0–4080	8.5	1.0	0–550

a Including children with zero parental gonadal dose.

b Sum of arithmetic means of individual stochastic doses from different exposure pathways.

**Table 7.** Cross-tabulation of paternal and maternal gonadal DOB-51 and DOB-38 doses from all exposure pathways^a,b^.

	Dose range (mGy)	Number of children with paternal gonadal DOB-38 dose in range							
		<3	3.0–9.99	10–29.9	30–99.9	100–299.9	300–999.9	1000+	Total
Number of children with paternal gonadal DOB-51 dose in range	<3.0	144	—	—	—	—	—	—	144
	3.0–9.99	—	31	1	—	—	—	—	32
	10–29.9	—	—	34	—	—	—	—	34
	30–99.9	—	—	—	32	1	1	—	34
	100–299.9	—	—	—	—	42	2	—	44
	300–999.9	—	—	—	—	—	45	1	46
	1000+	—	—	—	—	—	—	24	24
	Total	144	31	35	32	43	48	25	358
	Dose range (mGy)	Number of children with maternal gonadal DOB-38 dose in range							
		<3	3.0–9.99	10–29.9	30–99.9	100–299.9	300–999.9	1000+	Total
Number of children with maternal gonadal DOB-51 dose in range	<3.0	250	—	—	—	—	—	—	250
	3.0–9.99	—	55	—	—	—	—	—	55
	10–29.9	—	—	40	—	—	—	—	40
	30–99.9	—	—	—	10	—	—	—	10
	100–299.9	—	—	—	—	1	—	—	1
	300–999.9	—	—	—	—	—	2	—	2
	1000+	—	—	—	—	—	—	—	—
	Total	250	55	40	10	1	2	—	358

a For 358 children, including those with zero parental gonadal dose.

b Sum of arithmetic means of individual stochastic doses from different exposure pathways.

**Table 8.** Distribution of children according to paternal gonadal DOB-38 doses^a^ due to different exposure pathways.

	External irradiation during the cleanup mission^b^			External irradiation during residence in Pripyat^b^			Residential exposure^c^			Total		
Interval of gonadal dose (mGy)	*N* ^d^	%	Mean dose^e^ (mGy)	*N*	%	Mean dose (mGy)	*N*	%	Mean dose (mGy)	*N*	%	Mean dose (mGy)
<3.0	19	8.9	1.1	—	—	—	319	89.1	1.0	144	40.2	0.86
3.0–9.99	21	9.8	5.6	5	83.3	5.9	29	8.1	5.2	31	8.7	6.1
10–29.9	31	14.5	18	1	16.7	17	7	2.0	14	35	9.8	18
30–99.9	27	12.6	66	—	—	—	3	0.8	46	32	8.9	63
100–299.9	43	20.1	196	—	—	—	—	—	—	43	12.0	198
300–999.9	48	22.4	540	—	—	—	—	—	—	48	13.4	542
⩾1000	25	11.7	1746	—	—	—	—	—	—	25	7.0	1748
Entire study	214	100.0	376	6	100.0	7.7	358	100.0 2.0		358	100.0	227

a Arithmetic mean of individual stochastic doses.

b Including only children whose fathers were exposed to this pathway.

c Including children with zero paternal gonadal dose during residence.

d Number of children.

e Arithmetic mean for dose interval.

**Table 9.** Distribution of children according to maternal gonadal DOB-38 doses^a^ due to different exposure pathways.

	External irradiation during the cleanup mission^b^			External irradiation during residence in Pripyat^b^			Residential exposure^c^			Total		
Interval of gonadal dose (mGy)	*N* ^d^	%	Mean dose^e^ (mGy)	*N*	%	Mean dose (mGy)	*N*	%	Mean dose (mGy)	*N*	%	Mean dose (mGy)
<3.0	15	32.6	0.74	9	15.5	1.5	321	89.7	0.91	250	69.8	0.88
3.0–9.99	12	26.1	6.5	32	55.2	5.7	28	7.8	5.4	55	15.3	6.2
10–29.9	14	30.4	16	14	24.1	13	4	1.1	19	40	11.2	16
30–99.9	3	6.5	51	2	3.5	58	5	1.4	43	10	2.8	49
100–299.9	—	—	—	1	1.7	259	—	—	—	1	0.3	260
300–999.9	2	4.4	550	—	—	—	—	—	—	2	0.6	551
⩾1000	—	—	—	—	—	—	—	—	—	—	—	—
Entire study	46	100.0	34	58	100.0	13	358	100.0	2.1	358	100.0	8.5

a Arithmetic mean of individual stochastic doses.

b Including only children whose mothers were exposed to this pathway.

c Including children with zero maternal gonadal dose during residence.

d Number of children.

e Arithmetic mean for dose interval.

**Table 10.** Fathers’ and mothers’ gonadal DOB-38 doses^a,b^ due to external irradiation during the cleanup mission by category of cleanup workers.

	Father’s gonadal DOB-38 dose (mGy)				Mother’s gonadal DOB-38 dose (mGy)			
Category of cleanup workers	*N*	Mean	Median	Range	*N*	Mean	Median	Range
Victims of the accident	1	4080	—	—	—	—	—	—
Witnesses of the accident	—	—	—	—	—	—	—	—
Early liquidators	12	256	19	0.92–1300	1	8.0	—	—
Chornobyl NPP personnel	—	—	—	—	1	0.82	—	—
Sent to assist the Chornobyl NPP staff	2	543	543	18–1070	1	0.27	—	—
Staff of AC-605	2	746	746	416–1075	—	—	—	—
Staff of Kurchatov Institute	—	—	—	—	—	—	—	—
Military liquidators	59	183	90	0.11–1120	2	9.5	9.5	7.3–12
Sent on mission	13	91	6.7	0.063–728	6	3.2	0.10	0.013–18
Staff of combinat	2	50	50	11–88	1	1.2	—	—
Mixed^c^	92	538	295	1.8–3620	22	39	9.3	0.37–550
Entire study	183	389	152	0.063–1080	34	27	7.0	0.013–550

a Arithmetic mean of individual stochastic gonadal doses.

b Doses are given for fathers and mothers, not for children. If a father or mother has two or three children, the highest paternal dose was included in the table.

c Mixed refers to a set of liquidators who worked at the Chornobyl site several times as members of different categories.

**Table 11.** Distributions of the GSDs attached to the paternal and maternal individual stochastic gonadal DOB-38 doses^a^.

	External irradiation during the cleanup mission			External irradiation during residence in Pripyat			External irradiation during residence			Ingestion of Cs isotopes during residence		
GSD interval	*N* ^b^	%	Mean dose^c^ (mGy)	*N*	%	Mean dose (mGy)	*N*	%	Mean dose (mGy)	*N*	%	Mean dose (mGy)
<1.3	5	1.9	59	—	—	—	418	59.0	0.95	89	12.6	0.41
1.3–1.49	51	19.6	332	8	12.5	5.9	290	40.9	2.0	603	85.2	0.63
1.5–1.99	139	53.5	250	40	62.5	7.0	1	0.1	26	16	2.2	0.75
2–2.49	43	16.5	314	8	12.5	11	—	—	—	—	—	—
2.5–2.99	19	7.3	611	8	12.5	49	—	—	—	—	—	—
3–3.49	1	0.4	1336	—	—	—	—	—	—	—	—	—
⩾3.5	2	0.8	1809	—	—	—	—	—	—	—	—	—
Entire study	260	100.0	316	64	100.0	13	709	100.0	1.4	710	100.0	0.60

a Including only children with non-zero parental dose due to the given exposure pathway.

b Number of children.

c Arithmetic mean for GSD interval.
