# Supplementary material for: Estimation of radiation gonadal doses for the American–Ukrainian trio study of parental irradiation in Chornobyl cleanup workers and evacuees and germline mutations in their offspring
Source: J Radiol Prot. Author manuscript; Available in PMC 2022 Nov 1. (PMC9426296; doi:10.1088/1361-6498/abf0f4)
Supplement: Appendix 3 [file NIHMS1830840-supplement-Appendix_3.pdf]

Appendix 3. Dosimetry questionnaire for residence at locations other than Pripyat

Study of parental irradiation of Ukrainian clean-up workers and evacuees and germline mutations in their off spring (trio study)
Dosimetry Questionnaire - Interview of father or mother for residential exposure

Subject's ID | Bar Code Label

Date of interview___/___/201 _   *Interviewer's last name* |_|_|_|_|_|_|_|_|_|_|_|_|_|_|_|

Time of interview beginning: ____:____

Section I **General information on subject**

1. **What is your last name?**
|_|_|_|_|_|_|_|_|_|_|_|_|_|_|_|_|_|_|_|_|

2. **What is your first name?**
|_|_|_|_|_|_|_|_|_|_|_|_|_|_|_|_|_|_|_|_|

3. **What is your patronymic name?**
|_|_|_|_|_|_|_|_|_|_|_|_|_|_|_|_|_|_|_|_|

4. **Is your last name the same as it was at the time of the Chornobyl accident in 1986?**
1 ❑ yes *(go to 5)*   2 ❑ no *(go to 4a)*
4a. **What was your last name at the time of the Chornobyl accident in 1986?**
|_|_|_|_|_|_|_|_|_|_|_|_|_|_|_|_|_|_|_|_|

5. **What is your date of birth?**
|_|_| *Day* |_|_| *Month* |1|9|_|_| *Year*

Section II. **My next questions are about the place where you were on 26 April, 1986 at the time of the Chornobyl accident. Tell me about the exact place where you were at that time, that was not your permanent residence.**

6. **In which settlement were you on April 26, 1986 at the time of the Chornobyl accident?**

|___|___|___|___|___|___|___|___|___|___|___|___|___|___|___|___|___|___|___|___|___|___|___|___|___|___|___|___|___|

*(if respondent was in Pripyat-town or Janov, complete attachment-questionnaire for residents of Pripyat-town or Janov. After you complete questionnaire for residents of Pripyat-town or Janov (**Appendix 2**), continue with question 7)*

(if respondent was in a city, other than Pripyat-town or Janov go to 15b)

9 ❑ do not remember

6a. **In which selsovet was this settlement?**

|___|___|___|___|___|___|___|___|___|___|___|___|___|___|___|___|___|___|___|___|___|___|___|___|___|___|___|___|___|

9 ❑ do not remember

6b. **In which rayon was this settlement?**

|___|___|___|___|___|___|___|___|___|___|___|___|___|___|___|___|___|___|___|___|___|___|___|___|___|___|___|___|___|

9 ❑ do not remember

6c. **In which oblast was this settlement?**

|___|___|___|___|___|___|___|___|___|___|___|___|___|___|___|___|___|___|___|___|___|___|___|___|___|___|___|___|___|

9 ❑ do not remember

6d. **Please tell me the name of the street**

street |___|___|___|___|___|___|___|___|___|___|___|___|___|___|___|___|___|___|___|___|___|___|___|___|___|

9 ❑ do not remember

6e. **Please tell me the building or house number, and the apartment number** *(if the dwelling was an apartment building)*

House # |___|___|___| *Building #* |___|___|___|___| *Apartment #* |___|___|___|___|

9 ❑ do not remember

Section *III.* **My next questions are about places where you lived during the time period between 26 April and date of birth of your child ____/____/____. Tell me please about the exact places where you did live or stay for more than ONE DAY between April 26 and June 30, 1986 and did live or stay for more than ONE MONTH during the time period between July 1, 1986 and ____/____/____**{*date of childbirth*}**.**

7. *(Fill in the answers from "a" through "g" in the table below by asking all of the following questions for each place)*
   a. **Where did you live or stay for more than ONE DAY between April 26 and June 30, 1986 and did live or stay for more than ONE MONTH during the time period between July 1, 1986 and ____/____/____** {*date of childbirth*}**? What was the name of the first** *(next)* **settlement where you lived or stayed?** *(Fill in the answers from "a" through "g" in table below for every settlement where you lived or stayed)*
   b. **In which rayon?**
   c. **In which oblast?**
   d. **What was the purpose of your staying in this settlement? Residence (1), evacuation (2), relocation (3); visit relatives (4); other (5)** *(if respondent does not remember insert code «9»)*
   e. **On what date did you arrive at this place?** *(Show calendar - Card #1)*
   f. **On what date did you depart from this place?**
   g. **In what type of building did you stay? wooden (1), brick or block single-storey (2), brick or concrete many-storeys (3), other material (4)** *(if respondent does not remember insert code «9»)*

   {If the respondent does not remember the date of departure or arrival from/ to a settlement, ask about month and year}

#	a. Settlement	b.Rayon	c. Oblast	d. Purpose of stay	e. Arrival	f. Departure	g.Const-ruction material
					Date	Date	
1					___/___/___	___/___/___	
2					___/___/___	___/___/___	
3					___/___/___	___/___/___	
4					___/___/___	___/___/___	
5					___/___/___	___/___/___	
6					___/___/___	___/___/___	
7					___/___/___	___/___/___	
8					___/___/___	___/___/___	
9					___/___/___	___/___/___	
10					___/___/___	___/___/___	

Section IV. **My next questions are about your consumption of milk, dairy products, potatoes, root vegetables, and meat.**

8. **Tell me about your consumption of milk, dairy products, root vegetables, and meat in all the settlements where you resided.**

Enter the name of first settlement from the table 7 into column "a" and ask a question from the column "b". In column "c" mark if respondent consumed milk ***(cow, goat)****, dairy products, meat (pork, poultry), potatoesand root vegetables in this settlement. Ask questions from columns "c", "d", and "e" separately for each type of milk and for entire amount of dairy products without specification.* *Milk consumption is measured in glasses and dairy products,meat, potatoes, and root vegetables – in grams.*

#	a. **Settlement**	b. **Milk/Milk products**	c. **Did you consume** *(milk, dairy products, meat, potatoes)* **in** *(Settlement)***?**			d. **How often did you consume** *(milk, dairy products, meat, potatoes)***?**				e. **What amount of milk, dairy products, meat, potatoes or root vegetables did you typically consume per day** ***(when you consumed it)*****?**					
			**Yes**	**No** *(go to next foodstuff or next settlement)*	**Do not remember** *(go to next foodstuff or next settlement)*	**Every Day**	**Few times per week**	**One time per week or less**	Do not remember	**< 1 glass of milk (<50 g of dairy product, meat, vegetables)**	**1 glass of milk (50 g of dairy product, meat, vegetables)**	**2 glasses of milk (100 g of dairy products, meat, vegetables)**	**3 glasses of milk (200 g of dairy products, meat, vegetable)**	**Other quantity (glasses, g/day)**	Do not reme-mber
		Private cow milk	1 ❑	2 ❑	9 ❑	1 ❑	2 ❑	3 ❑	9 ❑	1 ❑	2 ❑	3 ❑	4 ❑		9 ❑
		Shop milk	1 ❑	2 ❑	9 ❑	1 ❑	2 ❑	3 ❑	9 ❑	1 ❑	2 ❑	3 ❑	4 ❑		9 ❑
1		Dairy products	1 ❑	2 ❑	9 ❑	1 ❑	2 ❑	3 ❑	9 ❑	1 ❑	2 ❑	3 ❑	4 ❑		9 ❑
		Meat (pork, poultry)	1 ❑	2 ❑	9 ❑	1 ❑	2 ❑	3 ❑	9 ❑	1 ❑	2 ❑	3 ❑	4 ❑		9 ❑
		Potatoes, root vegetables	1 ❑	2 ❑	9 ❑	1 ❑	2 ❑	3 ❑	9 ❑	1 ❑	2 ❑	3 ❑	4 ❑		9 ❑
		Private cow milk	1 ❑	2 ❑	9 ❑	1 ❑	2 ❑	3 ❑	9 ❑	1 ❑	2 ❑	3 ❑	4 ❑		9 ❑
		Shop milk	1 ❑	2 ❑	9 ❑	1 ❑	2 ❑	3 ❑	9 ❑	1 ❑	2 ❑	3 ❑	4 ❑		9 ❑
2		Dairy products	1 ❑	2 ❑	9 ❑	1 ❑	2 ❑	3 ❑	9 ❑	1 ❑	2 ❑	3 ❑	4 ❑		9 ❑
		Meat (pork, poultry)	1 ❑	2 ❑	9 ❑	1 ❑	2 ❑	3 ❑	9 ❑	1 ❑	2 ❑	3 ❑	4 ❑		9 ❑
		Potatoes, root vegetables	1 ❑	2 ❑	9 ❑	1 ❑	2 ❑	3 ❑	9 ❑	1 ❑	2 ❑	3 ❑	4 ❑		9 ❑
		Private cow milk	1 ❑	2 ❑	9 ❑	1 ❑	2 ❑	3 ❑	9 ❑	1 ❑	2 ❑	3 ❑	4 ❑		9 ❑
		Shop milk	1 ❑	2 ❑	9 ❑	1 ❑	2 ❑	3 ❑	9 ❑	1 ❑	2 ❑	3 ❑	4 ❑		9 ❑
3		Dairy products	1 ❑	2 ❑	9 ❑	1 ❑	2 ❑	3 ❑	9 ❑	1 ❑	2 ❑	3 ❑	4 ❑		9 ❑
		Meat (pork, poultry)	1 ❑	2 ❑	9 ❑	1 ❑	2 ❑	3 ❑	9 ❑	1 ❑	2 ❑	3 ❑	4 ❑		9 ❑
		Potatoes, root vegetables	1 ❑	2 ❑	9 ❑	1 ❑	2 ❑	3 ❑	9 ❑	1 ❑	2 ❑	3 ❑	4 ❑		9 ❑
		Private cow milk	1 ❑	2 ❑	9 ❑	1 ❑	2 ❑	3 ❑	9 ❑	1 ❑	2 ❑	3 ❑	4 ❑		9 ❑
4		Shop milk	1 ❑	2 ❑	9 ❑	1 ❑	2 ❑	3 ❑	9 ❑	1 ❑	2 ❑	3 ❑	4 ❑		9 ❑
		Dairy products	1 ❑	2 ❑	9 ❑	1 ❑	2 ❑	3 ❑	9 ❑	1 ❑	2 ❑	3 ❑	4 ❑		9 ❑
		Meat (pork, poultry)	1 ❑	2 ❑	9 ❑	1 ❑	2 ❑	3 ❑	9 ❑	1 ❑	2 ❑	3 ❑	4 ❑		9 ❑

		Potatoes, root vegetables	1 ❑	2 ❑	9 ❑	1 ❑	2 ❑	3 ❑	9 ❑	1 ❑	2 ❑	3 ❑	4 ❑		9 ❑
		Private cow milk	1 ❑	2 ❑	9 ❑	1 ❑	2 ❑	3 ❑	9 ❑	1 ❑	2 ❑	3 ❑	4 ❑		9 ❑
		Shop milk	1 ❑	2 ❑	9 ❑	1 ❑	2 ❑	3 ❑	9 ❑	1 ❑	2 ❑	3 ❑	4 ❑		9 ❑
5		Dairy products	1 ❑	2 ❑	9 ❑	1 ❑	2 ❑	3 ❑	9 ❑	1 ❑	2 ❑	3 ❑	4 ❑		9 ❑
		Meat (pork, poultry)	1 ❑	2 ❑	9 ❑	1 ❑	2 ❑	3 ❑	9 ❑	1 ❑	2 ❑	3 ❑	4 ❑		9 ❑
		Potatoes, root vegetables	1 ❑	2 ❑	9 ❑	1 ❑	2 ❑	3 ❑	9 ❑	1 ❑	2 ❑	3 ❑	4 ❑		9 ❑
		Private cow milk	1 ❑	2 ❑	9 ❑	1 ❑	2 ❑	3 ❑	9 ❑	1 ❑	2 ❑	3 ❑	4 ❑		9 ❑
		Shop milk	1 ❑	2 ❑	9 ❑	1 ❑	2 ❑	3 ❑	9 ❑	1 ❑	2 ❑	3 ❑	4 ❑		9 ❑
6		Dairy products	1 ❑	2 ❑	9 ❑	1 ❑	2 ❑	3 ❑	9 ❑	1 ❑	2 ❑	3 ❑	4 ❑		9 ❑
		Meat (pork, poultry)	1 ❑	2 ❑	9 ❑	1 ❑	2 ❑	3 ❑	9 ❑	1 ❑	2 ❑	3 ❑	4 ❑		9 ❑
		Potatoes, root vegetables	1 ❑	2 ❑	9 ❑	1 ❑	2 ❑	3 ❑	9 ❑	1 ❑	2 ❑	3 ❑	4 ❑		9 ❑
		Private cow milk	1 ❑	2 ❑	9 ❑	1 ❑	2 ❑	3 ❑	9 ❑	1 ❑	2 ❑	3 ❑	4 ❑		9 ❑
		Shop milk	1 ❑	2 ❑	9 ❑	1 ❑	2 ❑	3 ❑	9 ❑	1 ❑	2 ❑	3 ❑	4 ❑		9 ❑
7		Dairy products	1 ❑	2 ❑	9 ❑	1 ❑	2 ❑	3 ❑	9 ❑	1 ❑	2 ❑	3 ❑	4 ❑		9 ❑
		Meat (pork, poultry)	1 ❑	2 ❑	9 ❑	1 ❑	2 ❑	3 ❑	9 ❑	1 ❑	2 ❑	3 ❑	4 ❑		9 ❑
		Potatoes, root vegetables	1 ❑	2 ❑	9 ❑	1 ❑	2 ❑	3 ❑	9 ❑	1 ❑	2 ❑	3 ❑	4 ❑		9 ❑
		Private cow milk	1 ❑	2 ❑	9 ❑	1 ❑	2 ❑	3 ❑	9 ❑	1 ❑	2 ❑	3 ❑	4 ❑		9 ❑
		Shop milk	1 ❑	2 ❑	9 ❑	1 ❑	2 ❑	3 ❑	9 ❑	1 ❑	2 ❑	3 ❑	4 ❑		9 ❑
8		Dairy products	1 ❑	2 ❑	9 ❑	1 ❑	2 ❑	3 ❑	9 ❑	1 ❑	2 ❑	3 ❑	4 ❑		9 ❑
		Meat (pork, poultry)	1 ❑	2 ❑	9 ❑	1 ❑	2 ❑	3 ❑	9 ❑	1 ❑	2 ❑	3 ❑	4 ❑		9 ❑
		Potatoes, root vegetables	1 ❑	2 ❑	9 ❑	1 ❑	2 ❑	3 ❑	9 ❑	1 ❑	2 ❑	3 ❑	4 ❑		9 ❑
		Private cow milk	1 ❑	2 ❑	9 ❑	1 ❑	2 ❑	3 ❑	9 ❑	1 ❑	2 ❑	3 ❑	4 ❑		9 ❑
		Shop milk	1 ❑	2 ❑	9 ❑	1 ❑	2 ❑	3 ❑	9 ❑	1 ❑	2 ❑	3 ❑	4 ❑		9 ❑
9		Dairy products	1 ❑	2 ❑	9 ❑	1 ❑	2 ❑	3 ❑	9 ❑	1 ❑	2 ❑	3 ❑	4 ❑		9 ❑
		Meat (pork, poultry)	1 ❑	2 ❑	9 ❑	1 ❑	2 ❑	3 ❑	9 ❑	1 ❑	2 ❑	3 ❑	4 ❑		9 ❑
		Potatoes, root vegetables	1 ❑	2 ❑	9 ❑	1 ❑	2 ❑	3 ❑	9 ❑	1 ❑	2 ❑	3 ❑	4 ❑		9 ❑
		Private cow milk	1 ❑	2 ❑	9 ❑	1 ❑	2 ❑	3 ❑	9 ❑	1 ❑	2 ❑	3 ❑	4 ❑		9 ❑
		Shop milk	1 ❑	2 ❑	9 ❑	1 ❑	2 ❑	3 ❑	9 ❑	1 ❑	2 ❑	3 ❑	4 ❑		9 ❑
10		Dairy products	1 ❑	2 ❑	9 ❑	1 ❑	2 ❑	3 ❑	9 ❑	1 ❑	2 ❑	3 ❑	4 ❑		9 ❑
		Meat (pork, poultry)	1 ❑	2 ❑	9 ❑	1 ❑	2 ❑	3 ❑	9 ❑	1 ❑	2 ❑	3 ❑	4 ❑		9 ❑
		Potatoes, root vegetables	1 ❑	2 ❑	9 ❑	1 ❑	2 ❑	3 ❑	9 ❑	1 ❑	2 ❑	3 ❑	4 ❑		9 ❑

8f. **Did you consumed fresh or dried mushrooms?**

1 ❑ yes 2 ❑ no 9 ❑ do not remember

Section V. **Assessment of the interview**

The completeness and accuracy of answers given by the respondent as estimated by the interviewer is:
1 ❑ good 2 ❑ satisfactory 3 ❑ poor

Time of interview end: ____:____

Interviewer's comments:

____
____
____
____
____
____
____
____
____
____

❑ Questionnaire was entered to DB Date: _ _ / _ _ /201 _ Operator: ________________________________

Name of quality control expert: |_|_|_|_|_|_|_|_|_|_|_|_|_|_|_|_|_| Date of control _ _ / _ _ /201 _
