# Supplementary material for: Estimation of radiation gonadal doses for the American–Ukrainian trio study of parental irradiation in Chornobyl cleanup workers and evacuees and germline mutations in their offspring
Source: J Radiol Prot. Author manuscript; Available in PMC 2022 Nov 1. (PMC9426296; doi:10.1088/1361-6498/abf0f4)
Supplement: Appendix 1 [file NIHMS1830840-supplement-Appendix_1.pdf]

Appendix 1. Dosimetry questionnaire for mission as Chornobyl cleanup worker

Subject's identification number: |_| |_|_|_|_|

STUDY OF PARENTAL IRRADIATION OF UKRAINIAN CLEAN-UP WORKERS AND EVACUEES AND GERMLINE MUTATIONS IN THEIR OFF SPRING (TRIO STUDY)

Subject Questionnaire – Cleanup worker

Information on the liquidator

0.1 Last name ________________________________

0.2 First name ________________________________

0.3 Patronymic name ________________________________

0.4 Date of birth day |__|__| month |__|__| 19|__|__|

0.5 Type of document used for proof of identity

1 ☐ passport
2 ☐ military passport
3 ☐ other, specify ____________

____

Serial number ________________________________

Number |__|__|__|__|__|__|__|__|

0.6 Home address

Resident of

1 ☐ city / town
2 ☐ rural settlement
3 ☐ other, specify

____

ZIP code |__|__|__|__|__|__|

oblast, region ________________________________

raion ________________________________

if in city:

city ________________________________

street ________________________________

street # |__|__|__|

apartment # |__|__|__|

if in countryside:

agricultural sovet ________________________________

settlement ________________________________

0.7 Telephone number: at work |__|__|__|__|__|__|__|

at home |__|__|__|__|__|__|__|

Last, first and patronymic names of interviewer ____

Date of interview: day |__|__| month |__|__| year 20|__|__|

Time interview began: hours |__|__| minutes |__|__|

Location of interview *(check one).*

1 ☐ polyclinic
2 ☐ other, specify ____

1. Some information on the liquidator

At first I would like to ask you to answer general questions and then to show me a document proving your liquidator's status.

1.1 Please show me a document proving your liquidator status.

a. *If a document was given, write down the following information:*

Type of document	Serial number, number, date of issue	Name of organisation that issued the document
______________________<br>______________________	|__|__|__| -<br>|__|__|__|__|__|__|__|__|__| |__|__|/|__|__|/19|__|__|	________________________________<br>________________________________
______________________<br>______________________	|__|__|__| -<br>|__|__|__|__|__|__|__|__|__| |__|__|/|__|__|/19|__|__|	________________________________<br>________________________________
______________________<br>______________________	|__|__|__| -<br>|__|__|__|__|__|__|__|__|__| |__|__|/|__|__|/19|__|__|	________________________________<br>________________________________

b. If the document is not available (lost, etc.), please tell me *(write "don't remember", if applicable):*

the reason why it is not available ____

the type of document ____

the organization that issued it ____

____

1.2 How many times were you sent on mission to the 30-km zone *(check one)?*

1 ☐ once
3 ☐ three times
2 ☐ twice
4 ☐ more, specify ____________________

The following sections (2-4) should be completed for each liquidator's mission to the 30-km zone separately. After completion of sections 2-4 for the first mission, complete the same sections in the additional sheets for the following missions, if the liquidator was sent to the 30-km zone several times. If he doesn't remember the date you are asking about, enter 99 into the box for the year (if the year is not remembered) and enter 99 into the box for the month (if the month is not remembered. If the liquidator doesn't remember the day, ask him if that day was in the beginning, in the middle or at the end of the month and then enter the letter "н" - for the beginning of the month, the letter "c" - for the middle of the month or the letter "к"- for the end of the month.

2. Information on the first mission to the 30-km zone

Now I am going to ask you questions on the reason of your participation in activities in the 30-km zone and will ask you to show me documents confirming the period of time spent in the 30-km zone (if available). If there were several missions, please try to remember all of them and to describe the working conditions in the zone and living conditions outside the 30-km zone.

2.1 Please tell me which organization sent you to the 30-km zone *(check one)*?

1 ☐ regular army
2 ☐ military reserve office (komisariyat)
3 ☐ organization subordinated to the Nuclear Industry Ministry (including NPP)
4 ☐ Ministry of internal Affairs/KGB
5 ☐ other

2.2 Please give me the exact name and location of that organization at the time you were sent to the 30-km zone:

name ______________________________

oblast ______________________________

city/settlement ______________________________

If name has changed, please give the new name ______________________________

2.3 Please tell me when you started your mission

|__|__| / |__|__| / 19|__|__|

2.4 Please tell me when you ended your mission

|__|__| / |__|__| / 19|__|__|

2.5 Do you have an official document to confirm the dates of your mission in the 30-km zone (*check one*)?

1 ☐ yes
2 ☐ no

If “yes” please show it to me.

If the document was shown, please write down the following information:

Type of document	Time spent in the 30-km zone
______________	from |__|__| / |__|__| / 19|__|__| to |__|__| / |__|__| / 19|__|__|
______________	from |__|__| / |__|__| / 19|__|__| to |__|__| / |__|__| / 19|__|__|
______________	from |__|__| / |__|__| / 19|__|__| to |__|__| / |__|__| / 19|__|__|
______________	from |__|__| / |__|__| / 19|__|__| to |__|__| / |__|__| / 19|__|__|

2.6 Please tell me the name of the organization to which you were subordinated during your mission to the 30-km zone *(if not indicated before)*.

____

2.7 Could you show me an official document indicating the radiation dose that you received (*check one*)?

1 ☐ yes
2 ☐ no

If "no" please indicate why:
1 ☐ lost
2 ☐ forgotten at home
3 ☐ other, specify ___________________________

If "yes" please show it to me. *(If the document was shown, or if the liquidator remembers the contents of the document, please write down the following information):*

Type of document, organization	Serial number, number, date of issue	Period of exposure, dose, unit	Is it a cumulative dose?
___________________<br>___________________<br>___________________	|__|__|__| -<br>|__|__|__|__|__|__|__|__| |__|__|/|__|__|/19|__|__|	from |__|__| / |__|__| / 19|__|__| to |__|__| / |__|__| / 19|__|__| dose |__|__|__|__|__| _____	1 ☐ yes<br>2 ☐ no<br>9 ☐ don't know
___________________<br>___________________<br>___________________	|__|__|__| -<br>|__|__|__|__|__|__|__|__| |__|__|/|__|__|/19|__|__|	from |__|__| / |__|__| / 19|__|__| to |__|__| / |__|__| / 19|__|__| dose |__|__|__|__|__| _____	1 ☐ yes<br>2 ☐ no<br>9 ☐ don't know
___________________<br>___________________<br>___________________	|__|__|__| -<br>|__|__|__|__|__|__|__|__| |__|__|/|__|__|/19|__|__|	from |__|__| / |__|__| / 19|__|__| to |__|__| / |__|__| / 19|__|__| dose |__|__|__|__|__| _____	1 ☐ yes<br>2 ☐ no<br>9 ☐ don't know

2.8 Did you work shifts (*check one)*?

1 ☐ yes
2 ☐ no
3 ☐ both, indicate dates when worked shifts   from |__|__| / |__|__| / 19|__|__|
to |__|__| / |__|__| / 19|__|__|
9 ☐ don't know

2.9 Please tell me where you usually worked in the 30-km zone and what proportion of your time *(percentage)* you spent in the following conditions *(check all that apply)*

1 ☐ outside buildings and vehicles (outdoor)
  1 ☐ yes, specify proportion of time _______%
  2 ☐ no
  9 ☐ don't remember
2 ☐ inside buildings
  1 ☐ yes, specify proportion of time _______%
  2 ☐ no
  9 ☐ don't remember
3 ☐ inside a vehicle (for example, car)
  1 ☐ yes, specify proportion of time _______%

2 ☐ no
9 ☐ don't remember
4 ☐ other, specify ______________
1 ☐ yes, specify proportion of time _______%
2 ☐ no
9 ☐ don't remember
9 ☐ don't remember where worked

2.10 Please tell me – using the list of settlements – the main areas where you worked, as well as the following information *(interviewer should write down all the main settlements, one on each line).*

Settlement, raion	Date when started day/month/year	Duration (days)	Average number of hours per day
a ______________________________	|__|__|/|__|__| / 19|__|__|	|__|__|__|	|__|__|
b ______________________________	|__|__|/|__|__| / 19|__|__|	|__|__|__|	|__|__|
c ______________________________	|__|__|/|__|__| / 19|__|__|	|__|__|__|	|__|__|
d______________________________	|__|__|/|__|__| / 19|__|__|	|__|__|__|	|__|__|
e ______________________________	|__|__|/|__|__| / 19|__|__|	|__|__|__|	|__|__|

2.11 Please tell me – using the list of settlements – the main areas where you lived or the settlements closest to your place of stay, as well as the following information *(interviewer should write down all the main settlements, one per line).*

Settlement, raion	Date when started to live there, duration (days) and average number of hours per day	What was the type of dwelling where you mainly lived?
a ____________________<br>____________________	|__|__| / |__|__| / 19|__|__| |__|__|__| days<br>|__|__| hours / day	1 ☐ tent<br>2 ☐ wooden<br>3 ☐ brick or concrete block<br>4 ☐ other, specify____________<br>9 ☐ don't remember
b ____________________<br>____________________	|__|__| / |__|__| / 19|__|__| |__|__|__| days<br>|__|__| hours / day	1 ☐ tent<br>2 ☐ wooden<br>3 ☐ brick or concrete block<br>4 ☐ other, specify____________<br>9 ☐ don't remember
c ____________________<br>____________________	|__|__| / |__|__| / 19|__|__| |__|__|__| days<br>|__|__| hours / day	1 ☐ tent<br>2 ☐ wooden<br>3 ☐ brick or concrete block<br>4 ☐ other, specify____________<br>9 ☐ don't remember
d ____________________<br>____________________	|__|__| / |__|__| / 19|__|__| |__|__|__| days<br>|__|__| hours / day	1 ☐ tent<br>2 ☐ wooden<br>3 ☐ brick or concrete block<br>4 ☐ other, specify____________<br>9 ☐ don't remember

Settlement, raion	Date when started to live there, duration (days) and average number of hours per day	What was the type of dwelling where you mainly lived?
e ____________________<br>____________________	|__|__| / |__|__| / 19|__|__| |__|__|__| days<br>|__|__| hours / day	1 ☐ tent<br>2 ☐ wooden<br>3 ☐ brick or concrete block<br>4 ☐ other, specify____________<br>9 ☐ don't remember

2.12 Please tell me the reason why you left the 30-km zone after the mission *(check one).*

1 ☐ your dose was higher than the permissible level
2 ☐ your dose was equal to the permissible level
3 ☐ your mission was over
4 ☐ illness
5 ☐ other, specify ____
6 ☐ have not left, still working there
9 ☐ don't remember

3. Conditions of work in the 30-km zone during the first mission

The following questions concern the methods used for dosimetry, radiation protection measures (if applicable) and type of work undertaken during your mission to the 30-km zone.

3.1 Was your dose estimated *(check one)?*

1 ☐ yes
2 ☐ no
9 ☐ don't know

If "yes" please indicate the method and approximate time period when it was applied *(check one - "yes", "no" or "don't remember" for each method; if "yes" indicate requested information).* If your dose was estimated with a personal dosimeter, please indicate for each time period the number corresponding to the dosimeter you wore *(see photographs from the booklet).*

Method of estimation		Period	Number of dosimeter
With a personal dosimeter *(see photographs from the booklet)*	1 ☐ yes	from|__|__|/|__|__|/19|__|__|to|__|__|/|__|__|/19|__|__|	|__|__|
		from|__|__|/|__|__|/19|__|__|to|__|__|/|__|__|/19|__|__|	|__|__|
		from|__|__|/|__|__|/19|__|__|to|__|__|/|__|__|/19|__|__|	|__|__|
		from|__|__|/|__|__|/19|__|__|to|__|__|/|__|__|/19|__|__|	|__|__|
	2 ☐ no		
	9 ☐ don't remember		
By group dosimetry	1 ☐ yes	from|__|__|/|__|__|/19|__|__|to|__|__|/|__|__|/19|__|__|	
		from|__|__|/|__|__|/19|__|__|to|__|__|/|__|__|/19|__|__|	
		from|__|__|/|__|__|/19|__|__|to|__|__|/|__|__|/19|__|__|	
	2 ☐ no		
	9 ☐ don't remember		

Method of estimation		Period	Number of dosimeter
By itinerary	1 ☐ yes	from|__|__|/|__|__|/19|__|__|to|__|__|/|__|__|/19|__|__|	
		from|__|__|/|__|__|/19|__|__|to|__|__|/|__|__|/19|__|__|	
		from|__|__|/|__|__|/19|__|__|to|__|__|/|__|__|/19|__|__|	
	2 ☐ no		
	9 ☐ don't remember		

3.2 If you had a personal dosimeter, how often did you wear it *(check one)?*

1 ☐ all the time
2 ☐ only at work
3 ☐ sometimes at work
9 ☐ don't remember

3.3 Was the dosimeter regularly returned to the dosimetry service *(check one)?*

1 ☐ yes
2 ☐ no
9 ☐ don't remember

If "yes", please tell me how often *(check one)?*

1 ☐ daily
2 ☐ once a week
3 ☐ every two weeks
4 ☐ once a month
5 ☐ always when dosimetry service asked to return it
9 ☐ don't remember

3.4 Did you estimate your own radiation dose while working *(check one)?*

1 ☐ yes
2 ☐ no
9 ☐ don't remember

If "yes", what was your estimated dose? |__|__|__|__|__|__|

Indicate unit of estimated dose (*check one*):

1 ☐ rem
2 ☐ rad
3 ☐ R (Roentgen)
4 ☐ other, specify ____________________
9 ☐ unknown

What was your attitude to radiation exposure while staying in the 30-km zone? If you believe that your dose was higher than that received by your colleagues, explain why you think so.

____

____

____

3.5 Did you participate in any of the following activities *(check one of the boxes on each line)*?

Type of activity	Yes	No	Don't remember
Construction of sarcophagus on the industrial site of the Chornobyl NPP (ChNPP)	1 ☐	2 ☐	9 ☐
Removal of the radioactive fragments and pieces of graphite from the roofs or places close to the ventilation chimney	1 ☐	2 ☐	9 ☐
Decontamination of the rooms and equipment inside ChNPP buildings	1 ☐	2 ☐	9 ☐

Type of activity	Yes	No	Don't remember
Decontamination of industrial site and neighbourhood, including equipment outside ChNPP buildings	1 ☐	2 ☐	9 ☐
Dosimetry service (razvedka)	1 ☐	2 ☐	9 ☐
Decontamination of vehicles in PUSO (Points for strict sanitary clean-up)	1 ☐	2 ☐	9 ☐
Repair and servicing of ChNPP equipment	1 ☐	2 ☐	9 ☐
Other types of activity on the industrial site, specify ____________	1 ☐	2 ☐	9 ☐
Decontamination activities and burial of radioactive waste outside the industrial site	1 ☐	2 ☐	9 ☐
Construction of roads inside the 30-km zone	1 ☐	2 ☐	9 ☐
Working as a driver	1 ☐	2 ☐	9 ☐
Guarding of objects of ChNPP or inside the 30-km zone	1 ☐	2 ☐	9 ☐
Other types of activities outside the industrial site, specify ____________	1 ☐	2 ☐	9 ☐

3.6 Did you ever use any of the following protective measures while working in the 30-km zone *(check one of the boxes on each line)*?

Type of protective measure	Yes	No	Don't remember
Respirator or gas mask	1 ☐	2 ☐	9 ☐
Gloves	1 ☐	2 ☐	9 ☐
Protective glasses	1 ☐	2 ☐	9 ☐
Protective clothes	1 ☐	2 ☐	9 ☐
Lead apron	1 ☐	2 ☐	9 ☐
Vehicles with protective covering (armoured cars, lead sheets in helicopters)	1 ☐	2 ☐	9 ☐
Other, specify ____________	1 ☐	2 ☐	9 ☐

3.7 Did you work on the industrial site of ChNPP *(check one)?*

1 ☐ yes
2 ☐ no
9 ☐ don't remember

3.8 Please list the people who worked with you.

Last, first and patronymic names	Title

4. Description of the first episode of work during the first participation in the 30-km zone

In the following section I would like to ask you to remember in detail the work that you did. If you worked in the 30-km zone during the first days after the accident - at the end of April and beginning of May - please start from the episodes when you were most exposed to radiation.

4.1 Please tell me where you worked *(use the photographs, schemes and maps from Booklet - part B- as well as explanation of abbreviations- pp. 16-19 -, list of rooms - pp. 20-23- and of settlements -pp. 24-57 - from Booklet - part A. Check all that apply):*

- 1 ☐ on the industrial site of the ChNPP inside buildings *(complete section a)*
- 2 ☐ on the industrial site of the ChNPP on the roof of buildings *(complete section b)*
- 3 ☐ outside buildings at other locations of the industrial site of the CNPP *(complete section c)*
- 4 ☐ outside buildings and outside the industrial site of the CNPP *(complete section c)*
- 5 ☐ inside vehicles - in the 30-km zone, including the industrial site -*(complete section d)*
- 9 ☐ don't remember (*(go to question 4.2)*

a. <u>On the industrial site of the ChNPP inside buildings</u> *(if no, go to paragraph b. and c.)*:

a.1 Show me the building where you worked in *(use the schemes and photos from Booklet - part B -, and also explanation of abbreviations- pp. 16-19 - and list of rooms - pp. 20-23 - from Booklet - part A. Select one number and enter it).* |__| |__|__|__|__|__|__|__|

a.2 How long did it take you to walk to the place where you worked after entering the building *(enter duration in minutes)?* |__|__|__|

a.3 Did you pass more than two staircases on the way?

- 1 ☐ yes
- 2 ☐ no
- 9 ☐ don't remember

a.4 What was the size of the room you worked in *(enter in m^2)?* |__|__|__|

a.5 Were there any windows?

- 1 ☐ yes
- 2 ☐ no
- 9 ☐ don't remember

a.6 If "yes", were they covered with lead?

- 1 ☐ yes
- 2 ☐ no
- 9 ☐ don't remember

a.7 What was the colour of the walls *(check one)*?

1 ☐ white	5 ☐ green
2 ☐ grey	6 ☐ brown
3 ☐ gold	7 ☐ other, specify ________________________
4 ☐ silver	9 ☐ don't remember

a.8 Was the door thick *(check one)*?

- 1 ☐ yes
- 2 ☐ no
- 9 ☐ don't remember

a.9 What was the floor covered with *(check one)*?

1 ☐ plastic	4 ☐ wood
2 ☐ concrete	5 ☐ other, specify ________________________
3 ☐ tiles	9 ☐ don't remember

a.10 Was there large machinery in the room *(check one)*?

- 1 ☐ yes
- 2 ☐ no

9 ☐ don't remember

a.11 Give more details describing your working place in the building:

____

____

b. <u>On the industrial site of the ChNPP on the roof of buildings:</u>

b.1 On the roof of which building did you work *(use the schemes and photos from Booklet - part B -, select one number from the scheme or map - pp. 2-3 - and enter it)*? |__| |__|__|__|

b.2 Were there other buildings nearby *(check one)*?

1 ☐ yes
2 ☐ no
9 ☐ don't remember

If "yes" please enter the numbers of buildings from scheme-map of buildings and structures on the CHNPP plant grounds from booklet:

|__| |__|__|__| / |__| |__|__|__| / |__| |__|__|__|

b.3 Was the roof you worked on *(check one)*:

1 ☐ higher than other buildings?
2 ☐ lower than other buildings?
9 ☐ don't remember?

b.4 Did the roof have different levels (*check one*)?

1 ☐ yes
2 ☐ no
9 ☐ don't remember

b.5 What was the size of the roof? (*enter in* m^2) |__|__|__|

b.6 What material was the roof covered with *(check one)*?

1 ☐ concrete
2 ☐ asbestos sheets
3 ☐ wood
4 ☐ asphalt
5 ☐ other, specify ______________________
9 ☐ don't remember

b.7 Was the roof damaged near the place you worked (*check one*)?

1 ☐ yes
2 ☐ no
9 ☐ don't remember

b.8 Were there debris or waste materials near the place you worked *(check one*)?

1 ☐ yes
2 ☐ no
9 ☐ don't remember

b.9 Where was the staircase leading to the roof *(check one*)?

1 ☐ outside the building
2 ☐ in the building
9 ☐ don't remember

b.10 Give more details describing your working place on the roof:

____

____

c. <u>Outside buildings:</u>

c.1 If you worked on the industrial site of the ChNPP, but not on the roof of buildings, please indicate the number of building you worked nearby *(use the schemes and photos from Booklet and enter a number)* |__| |__|__|__|

c.2 If you worked outside on the industrial site of the ChNPP, please indicate the name of settlement (raion, oblast, etc.) were (or nearby) you worked *(use the maps and lists of settlements):*

____

c.3 Were there any of the following landmarks near the place you worked *(check all that apply)*?

- 1 ☐ buildings
- 2 ☐ electricity pylons
- 3 ☐ other, specify ________________________________
- 9 ☐ don't remember

c.4 What kind of machinery was nearby *(check all that apply)?*

- 1 ☐ bulldozers
- 2 ☐ dump trucks
- 3 ☐ excavators
- 4 ☐ cranes
- 5 ☐ other machinery, specify __________________
- 9 ☐ don't remember

c.5 What was under your feet *(check one)?*

- 1 ☐ earth
- 2 ☐ gravel
- 3 ☐ concrete
- 4 ☐ asphalt
- 5 ☐ sand
- 6 ☐ other, specify ________________________
- 9 ☐ don't remember

c.6 Were there procedures to reduce the dust level *(check one)?*

- 1 ☐ yes
- 2 ☐ no
- 9 ☐ don't remember

c.7 Give more details describing your working place:

____

____

d. <u>In vehicles (in the 30-km zone, including the industrial site):</u>:

d.1 Indicate the type of vehicle you worked in *(check one):*

- 1 ☐ bulldozer
- 2 ☐ bus
- 3 ☐ dump truck
- 4 ☐ car
- 5 ☐ tractor
- 6 ☐ crane
- 7 ☐ other, specify ________________________
- 9 ☐ don't remember

d.2 Did the vehicle have any protective covering *(check one)?*

- 1 ☐ yes
- 2 ☐ no
- 9 ☐ don't remember

If "yes" describe it: ____

d.3 Describe the routes that were followed: roads, settlements, production sites *(use the maps and lists of settlements):*____

____

4.2 Type of activities in the episode:

a. Please describe in detail the work that you did, and indicate what tools and devices were used:

____

____

____

b. How did you work *(check one)?*

- 1 ☐ in a group

2 ☐ alone
9 ☐ don't remember

c. Did you work in "mogilnik" (places where radioactive waste was buried) *(check one)?*
1 ☐ yes
2 ☐ no
9 ☐ don't remember

4.3 Commuting to the place of work in the episode:

a. What type of transportation did you use to commute back and forth to your working place *(check one)*?

1 ☐ BTR (armoured car)	5 ☐ car
2 ☐ bus	6 ☐ lorry
3 ☐ tractor	7 ☐ other, specify ________________
4 ☐ helicopter	9 ☐ don't remember

b. Please describe the route you followed to get to your working place *(use the maps and lists of settlements):*

From where you started ______________________________

to where you went ______________________________

landmarks along the route ______________________________

c. Did you change vehicles while commuting *(check one)*?
1 ☐ yes
2 ☐ no
9 ☐ don't remember

If "yes" specify where you did it ______________________________

d. How long did it take to get from the place where you lived to the place where you worked and back *(enter in minutes)*? |__|__|__|

e. Did you walk to your working place after getting out of the vehicle *(check one)*?
1 ☐ yes
2 ☐ no
9 ☐ don't remember

If "yes" please enter duration in minutes |__|__|__|

f. Please describe the route you followed to get back from your working place to the place you lived:

from where you started______________________________

to where you went ______________________________

landmarks along the route ______________________________

4.4 Meals and leisure in the episode

a. Where did you take your meals during working hours *(use maps and photos from Booklet - part B):*
1 ☐ outside a building *(enter number of closest building from the map or scheme or enter name of settlement)*: ______________________
2 ☐ in a building *(enter number of building from the map or scheme or enter name of settlement)*: ______________________
3 ☐ in a vehicle *(specify which)* ______________________
9 ☐ don't remember

b. How did you get to the place where you ate *(check one)*?
1 ☐ on foot
2 ☐ by transport
9 ☐ don't remember

If by transport, please indicate how long it took *(enter duration in minutes)*? |__|__|__|

and route ____

c. How long was your lunch break *(enter duration in minutes)*? |__|__|__|

d. Where did you rest during breaks *(use the maps and photos from Booklet and check on answer)*?

1 ☐ outside a building *(enter number of closest building from the map and scheme 2-3 or enter name of settlement)*: ____________________

2 ☐ in a building *(enter number of building from the map or scheme 2-3 or enter name of settlement)*: ____________________

3 ☐ in a vehicle *(specify which)* ____________________

9 ☐ don't remember

e. How did you get to the place where you rested (*check one*)?

1 ☐ on foot

2 ☐ by transport

9 ☐ don't remember

If by transport, please indicate how long it took *(enter duration in minutes)*? |__|__|__|

and route ____

f. How long did you rest during your working day *(enter duration in minutes)*? |__|__|__|

4.5 Time period of the episode: from |__|__|/|__|__|/19|__|__| to |__|__|/|__|__|/19|__|__|

Working days during the episode: *(circle the days that apply if the liquidator worked in April-May-June 1986, and circle the months if he worked later - starting from July 1986 until the end of 1987)*

April / May 1986						
M	*T*	*W*	*Th*	*F*	*S*	*S*
21	22	23	24	25	26	27
28	29	30	1	2	3	4
5	6	7	8	9	10	11
12	13	14	15	16	17	18
19	20	21	22	23	24	25
26	27	28	29	30	31	

June 1986						
M	*T*	*W*	*Th*	*F*	*S*	*S*
						1
2	3	4	5	6	8	8
9	10	11	12	13	14	15
16	17	18	19	20	21	22
23	24	25	26	27	28	29
30						

1986	1987	
Jul	Jan	Jul
Aug	Feb	Aug
Sep	Mar	Sep
Oct	Apr	Oct
Nov	May	Nov
Dec	Jun	Dec

4.6 Number of working days during the episode *(on average)*) |__|__|

4.7 Dosimetric control during the episode:

a. Did you change your clothes before work (*check one*)?

1 ☐ yes

2 ☐ no

9 ☐ don't remember

b. Did you start to work immediately upon arrival (*check one*)?

1 ☐ yes

2 ☐ no

9 ☐ don't remember

If "no" please enter duration of time before you started your work in minutes |__|__|__|

c. Did you pass through control points for checking radioactive contamination (*check one*)?

1 ☐ yes

2 ☐ no

9 ☐ don't remember

d. Did you pass through containers filled with potassium permanganate *(check one)*?

1 ☐ yes
2 ☐ no
9 ☐ don't remember

e. Did a dosimetrist control the radiation situation during your work or was there one nearby *(check one)*?

1 ☐ yes
2 ☐ no
9 ☐ don't remember

f. Did your supervisor communicate with the dosimetrist *(check one)*?

1 ☐ yes
2 ☐ no
9 ☐ don't remember

g. Was the dosimetrist from the military *(check one)*?

1 ☐ yes
2 ☐ no
9 ☐ don't remember

4.8 Your comments concerning the episode:

a. Do you think that the activities described above were well organised and under the control of a supervisor, dosimetrist, etc. *(check one)*?

1 ☐ yes
2 ☐ no
9 ☐ don't remember

b. What surprised you during your work?

____

____

c. Please give any comments concerning your activities during the episode.

____

____

5. Conclusion

Thank you very much for answering my questions. Please use the lines below for any additional comments you may have.

____

____

____

____

____

____

____

Time interview ended: hours |__|__| minutes |__|__|

6. Questions to the interviewer

6.1 Was respondent responsive *(check one)*?

1 ☐ no (was uninterested, reticent)
2 ☐ fairly cooperative and responsive
3 ☐ very cooperative, helpful

6.2 How well did the liquidator seem to remember the details of his work in the 30-km zone *(check one)?*

1 ☐ very well
2 ☐ well
3 ☐ fairly well
4 ☐ not well
5 ☐ not at all

6.3 If the liquidator answered that he had worked on the industrial site of the Chornobyl NPP, how confident are you about his answers *(please take into consideration how he described his work, proposed maps, schemes and photographs)*?

1 ☐ I am confident
2 ☐ I am not sure
3 ☐ I do not think that he worked on the industrial site of the Chornobyl NPP
8 ☐ liquidator answered that he didn't work on the industrial site
9 ☐ I don't know

explain, why do you think so ____

____

____

6.9 Please add any additional notes, comments on the liquidator's answers and your general comments on the interview. In particular, please provide further details and information, not covered in the questionnaire but received from the liquidator, if you find them to be relevant; please give all the details about liquidator's state which might have affected quality of his answers (such as: was he ill? did he have any problem? or was in a hurry during the interview?).

____

____

____

____

____

____

____

____

____

____

____

____

____

____
