# Supplementary material for: Estimation of radiation gonadal doses for the American–Ukrainian trio study of parental irradiation in Chornobyl cleanup workers and evacuees and germline mutations in their offspring
Source: J Radiol Prot. Author manuscript; Available in PMC 2022 Nov 1. (PMC9426296; doi:10.1088/1361-6498/abf0f4)
Supplement: Appendix 2 [file NIHMS1830840-supplement-Appendix_2.pdf]

Appendix 2. Dosimetry questionnaire for residence in Pripyat

Study of parental irradiation of Ukrainian clean-up workers and evacuees and germline mutations in their offspring (Trio study)

Subject's ID |__||__||__||__||__||__||__||__|

Respondent

1 ❑ father 2 ❑ mother

Last name of the Pripyat resident

|__|__|__|__|__|__|__|__|__|__|__|__|__|__|__|__|__|__|__|__|

Date of the interview / /201 *Interviewer's last name* |__|__|__|__|__|__|__|__|__|__|__|__|__|__|__|

2. I am going to show you a map of Pripyat town. What was your address at Pripyat?

2a. **Please tell me the name of the street**

street |__|__|__|__|__|__|__|__|__|__|__|__|__|__|__|__|__|__|__|__|__|__|__|__|__|

9 ❑ do not remember

2b. **Please tell me the building or house number, and the apartment number**

House # |__|__|__| *Building #* |__|__|__|__| *Apartment #* |__|__|__|__|

9 ❑ do not remember

3. Were you within the city or outside the city at the time of the Chernobyl accident on Apr 26, 1986?

1 ❑ in the city 2 ❑ outside the city. Exactly where?______________________________ 9 ❑ don't know

4. When did you learn about the accident? What date was it?

|__|__| April 9 ❑ don't know

4a. What time was it? *{ask about hours and enter minutes only if respondent can tell exact time}*

|__|__| hours |__|__| min 9 ❑ don't know

5. What precautionary activities were undertaken by your family after you learned about the accident?

1 ❑ limiting of time outside 2 ❑ closing windows 3 ❑ none 9 ❑ don't know

6. When were you evacuated from Pripyat? What date was it?

|__|__| April 9 ❑ don't know

7. **Please try to recall your/***{name}* **location by hours from the beginning of the accident until the evacuation. Indicate on the map where you/***{name}* **were at different periods of the day from the night of Apr 26 until the evacuation. Mark on the map your/*{name}* approximate route of relocation in the city until the evacuation** *{show the respondent the map of the city, give him/her a chance to mark a house and other attended places. Record in the appropriate cell of the table the section number of Pripyat city, where the respondent was; if the respondent was outside of the city, then make a special mark on the side of the map and write down the name of the settlement. Fill in the information for all the columns before the evacuation; after the evacuation leave a crossing line}*

Period of the day	Night	Morning		Afternoon			Evening		Night
Hours	From<br>2 am to 7 am	From<br>7 am 9 am	From<br>9 am to 11 am	From<br>11 am to 1 pm	From<br>1 pm to 3 pm	From<br>3 pm to 5 pm	From<br>5 pm to 7 pm	From<br>7 pm to 9 pm	From 9 pm<br>to до 2 am
7.1a. **On April 26, 1986 during *{day period, hours}* were you/***{name}* **mostly indoors or outdoors?**	1 ❑ indoors<br>2 ❑ outdoors<br>9 ❑ DN	1 ❑ indoors<br>2 ❑ outdoors<br>9 ❑ DN	1 ❑ indoors<br>2 ❑outdoors<br>9 ❑ DN	1 ❑ indoors<br>2 ❑outdoors<br>9 ❑ DN	1 ❑ indoors<br>2 ❑outdoors<br>9 ❑ DN	1 ❑ indoors<br>2 ❑outdoors<br>9 ❑ DN	1 ❑ indoors<br>2 ❑outdoors<br>9 ❑ DN	1 ❑ indoors<br>2 ❑outdoors<br>9 ❑ DN	1 ❑ indoors<br>2 ❑outdoors<br>9 ❑ DN
7.1b. **At what address (street / house #) did you stay** *{name}*? **If you were outside Pripyat, provide name of settlement where did you stay.**									
7.2a. **On April 27, 1986 during *{day period, hours}* were you/***{name}* **mostly indoors or outdoors?**	1 ❑ indoors<br>2 ❑ outdoors<br>9 ❑ DN	1 ❑ indoors<br>2 ❑ outdoors<br>9 ❑ DN	1 ❑ indoors<br>2 ❑outdoors<br>9 ❑ DN	1 ❑ indoors<br>2 ❑outdoors<br>9 ❑ DN	1 ❑ indoors<br>2 ❑outdoors<br>9 ❑ DN	1 ❑ indoors<br>2 ❑outdoors<br>9 ❑ DN	1 ❑ indoors<br>2 ❑outdoors<br>9 ❑ DN	1 ❑ indoors<br>2 ❑outdoors<br>9 ❑ DN	1 ❑ indoors<br>2 ❑outdoors<br>9 ❑ DN
7.2b. **At what address (street / house #) did you stay** *{name}*? **If you were outside Pripyat, provide name of settlement where did you stay.**									
7.3a. **On April 28, 1986 during *{day period, hours}* were you/***{name}* **mostly indoors or outdoors?**	1 ❑ indoors<br>2 ❑ outdoors<br>9 ❑ DN	1 ❑ indoors<br>2 ❑ outdoors<br>9 ❑ DN	1 ❑ indoors<br>2 ❑outdoors<br>9 ❑ DN	1 ❑ indoors<br>2 ❑outdoors<br>9 ❑ DN	1 ❑ indoors<br>2 ❑outdoors<br>9 ❑ DN	1 ❑ indoors<br>2 ❑outdoors<br>9 ❑ DN	1 ❑ indoors<br>2 ❑outdoors<br>9 ❑ DN	1 ❑ indoors<br>2 ❑outdoors<br>9 ❑ DN	1 ❑ indoors<br>2 ❑outdoors<br>9 ❑ DN
7.3b. **At what address (street / house #) did you stay** *{name}*? **If you were outside Pripyat, provide name of settlement where did you stay.**									

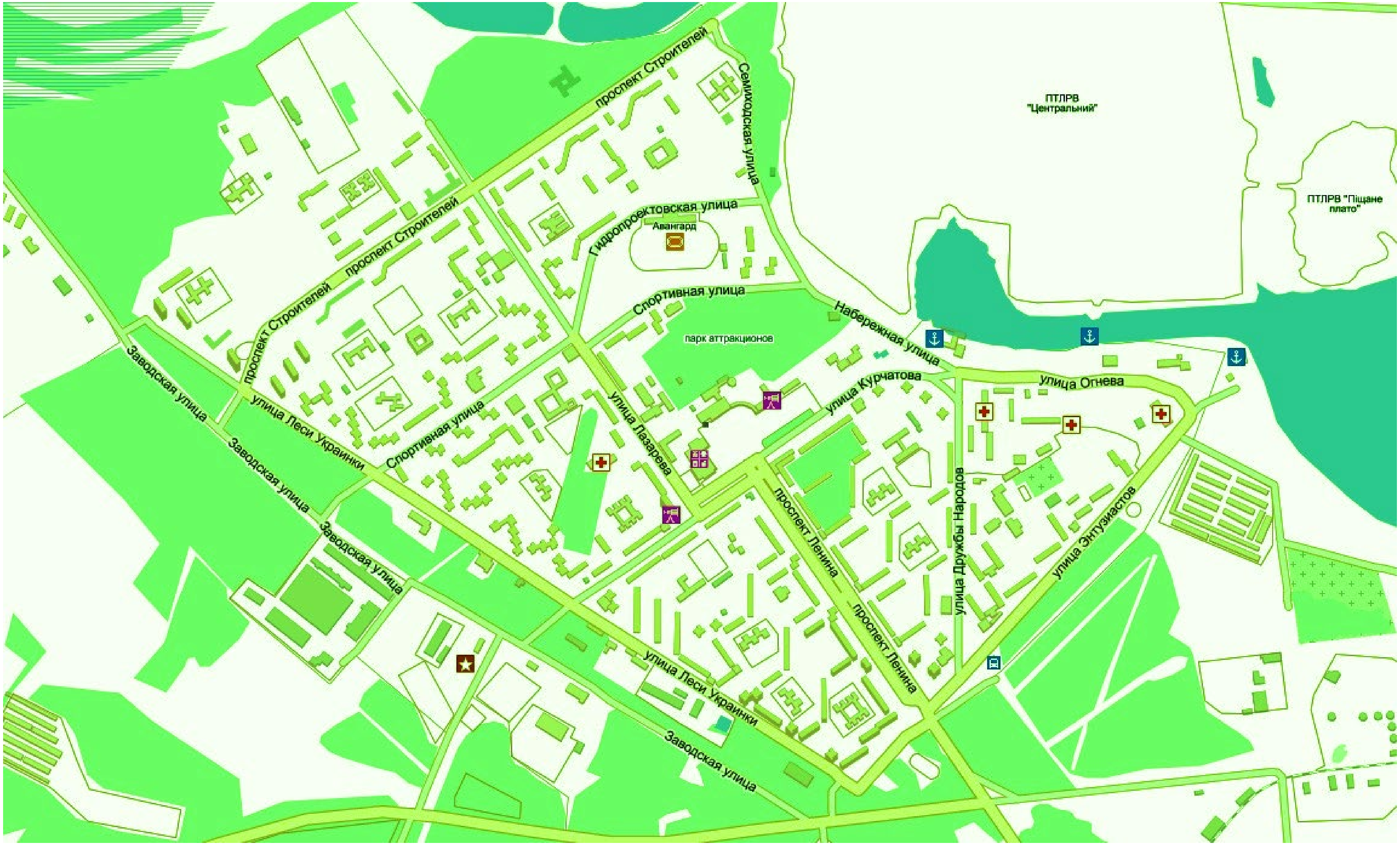
проспект Строителей
Семиходская улица
ПТЛРВ "Центральний"
ПТЛРВ "Піщане плато"
Гидропроектовская улица
Авангард
проспект Строителей
Спортивная улица
Набережная улица
парк аттракционов
улица Курчатова
улица Огнева
Заводская улица
улица Леси Украинки
Спортивная улица
улица Лазарева
проспект Ленина
улица Дружбы Народов
улица Энтузиастов
Заводская улица
Заводская улица
улица Леси Украинки
Заводская улица

Now I am going to ask you about the evacuation day in detail.
8. How were you/{name} evacuated from Pripyat?

1 ❑ by organized bus 2 ❑ independently by car 3 ❑ by train 4 ❑ other. Specify ____________________ 9 ❑ don't know *(go q.6q)*

9. What time did you/*{name}* **leave the house for evacuation?** *{enter minutes only if respondent can tell exact time}*

|__|__| hours |__|__| min 9 ❑ don't know

10. What time did the bus/*{car/train}* **leave on the road?** *{enter minutes only if respondent can tell exact time}*

|__|__| hours |__|__| min 9 ❑ don't know

11. What was the name of the place where you/*{name}* **arrived?**

|___|___|___|___|___|___|___|___|___|___|___|___|___|___|___|___|___|___|___|___|___|___|___|___|___|___|___|___|___|___| 9 ❑ don't know

11a. **In what raion was this settlement?**

|___|___|___|___|___|___|___|___|___|___|___|___|___|___|___|___|___|___|___|___|___|___|___|___|___|___|___|___|___|___| 9 ❑don't know

11b. **In what oblast/***{Republic}* **was this settlement?**

|___|___|___|___|___|___|___|___|___|___|___|___|___|___|___|___|___|___|___|___|___|___|___|___|___|___|___|___|___|___| 9 ❑don't know

12. What time did you/*{name}* **arrive at {***settlement***}?** *{enter minutes only if respondent can tell exact time}*

|__|__| hours |__|__| min (go to q.6s) 9 ❑ don't know

13. *{If respondent does not remember departure time from Pripyat, then ask following}* **How long did your trip last?** *{enter minutes only if respondent can tell exact time}*

|__|__| hours |__|__| min 9 ❑ don't know

Assessment of the interview

The completeness and accuracy of answers given by the respondent as estimated by the interviewer is:

1 ❑ good 2 ❑ satisfactory 3 ❑ poor

Time of interview end: ____:____

Interviewer's comments:

____

____

____

____

____

____

____

____

____

____

❑ Questionnaire was entered to DB Date: _ _ / _ _ /201 _ Operator: ________________________________

Name of quality control expert: |_|_|_|_|_|_|_|_|_|_|_|_|_|_|_|_|_| Date of control _ _ / _ _ /201 _
